# Subpopulations of neurons in the perirhinal cortex enable both modality-specific and modality-invariant recognition of objects

**DOI:** 10.1371/journal.pbio.3002713

**Published:** 2024-06-26

**Authors:** Heung-Yeol Lim, Inah Lee

**Affiliations:** Department of Brain and Cognitive Sciences, Seoul National University, Seoul, Korea; Institute of Science and Technology Austria, AUSTRIA

## Abstract

The perirhinal cortex (PER) supports multimodal object recognition, but how multimodal information of objects is integrated within the PER remains unknown. Here, we recorded single units within the PER while rats performed a PER-dependent multimodal object-recognition task. In this task, audiovisual cues were presented simultaneously (multimodally) or separately (unimodally). We identified 2 types of object-selective neurons in the PER: crossmodal cells, showing constant firing patterns for an object irrespective of its modality, and unimodal cells, showing a preference for a specific modality. Unimodal cells further dissociated unimodal and multimodal versions of the object by modulating their firing rates according to the modality condition. A population-decoding analysis confirmed that the PER could perform both modality-invariant and modality-specific object decoding—the former for recognizing an object as the same in various conditions and the latter for remembering modality-specific experiences of the same object.

## Abstract 2

Object recognition has mainly been studied using unimodal stimuli. This study shows that a subpopulation of neurons in the perirhinal cortex uses unimodal and multimodal sensory information both for flexible recognition of a multimodal object based on unimodal cues, and for episodic experience of the object.

## Introduction

Our brains can effortlessly integrate information from different sensory modalities to form a unified representation of the world [1,2]. This natural ability is also evident during object recognition, as one can quickly identify a music box by visually perceiving its distinctive appearance or hearing its original sound. The ability to recognize objects crossmodally has been reported not only in humans but also in nonhuman primates [3,4], rodents [5–7], dolphins [8], and even insects [9]. However, most studies on object recognition have neglected the multisensory nature of this process. Object recognition has been studied primarily using unimodal stimuli, such as visual stimuli [10–12], or including uncontrolled multimodal stimuli, such as 3D “junk” objects [13,14], without a specific goal of investigating multimodal processing. This tendency is also evident in studies of the perirhinal cortex (PER), a region well known to play a critical role in object recognition [15–20].

Findings from several studies have implied that the PER is engaged in “multimodal” object recognition. Anatomically, it has been shown that the PER receives inputs from areas that process diverse sensory modalities, including those from visual, auditory, olfactory, and somatosensory cortices [21–23]. In rodents, in particular, these areas are known to send monosynaptic inputs to the PER [22]. Experimental results further support the involvement of the PER in multimodal object recognition. In human functional magnetic resonance imaging (fMRI) studies in which subjects were presented visual-auditory or visual-tactile features that were either from the same (congruent) or different (incongruent) objects, activity within the PER was found to be greater when the 2 stimuli were congruent [24,25]. The necessity of the PER for multimodal object recognition has also been tested using crossmodal versions of a delayed nonmatch-to-sample task in nonhuman primates [4] and a spontaneous object-recognition task in rodents [5–7]. In these tasks, in which animals sampled an object using one sensory modality (e.g., tactile), and then were tested for retrieval of object information using an unused sensory modality (e.g., visual), lesioning or inactivating the PER resulted in performance deficits. These results indicate the involvement of the PER in multimodal object recognition, but the mechanisms underlying these functions remain largely unknown.

We hypothesized that the PER may support multisensory object recognition by integrating multimodal inputs from an object to form a unified representation of the object. Considering the associative nature of the PER [26–29], the region can be expected to integrate information from multiple sensations rather than processing it separately. Indeed, it has been shown that PER neurons do not represent individual sensory attributes separately in rats performing behavioral tasks using multimodal stimuli [30,31]. However, these studies have only reported neural correlates of behavioral responses or rewards associated with objects rather than actual information about the objects themselves. Accordingly, in the current study, we investigated how multimodal information is integrated to create a unified representation of an object while minimizing the influence of other task-related variables, such as behavioral response or reward outcome.

To test the abovementioned hypothesis, we developed a multimodal object-recognition task for rats employing visual and auditory cues. By requiring a nose-poke during object cue sampling, our task allowed a clear definition of sample and responding phases while observing their neural firing correlates in a temporally controlled manner. Our findings suggest that rats can recognize a familiar object (originally learned multimodally) almost immediately when cued by a unimodal sensory attribute alone (e.g., visual or auditory) without additional learning. However, inactivating the PER resulted in performance deficits in both multimodal and unimodal recognition conditions. Physiologically, we discovered that the selective firing pattern for an object was comparable regardless of the stimulus modality in most PER neurons. However, a significant proportion of neurons also showed a preference for a specific sensory modality during object information processing. A population-decoding analysis revealed that these subpopulations of neurons enabled both modality-specific and modality-invariant recognition of objects.

## Results

### Rats can perform multimodal object-recognition task

**Fig 1:**
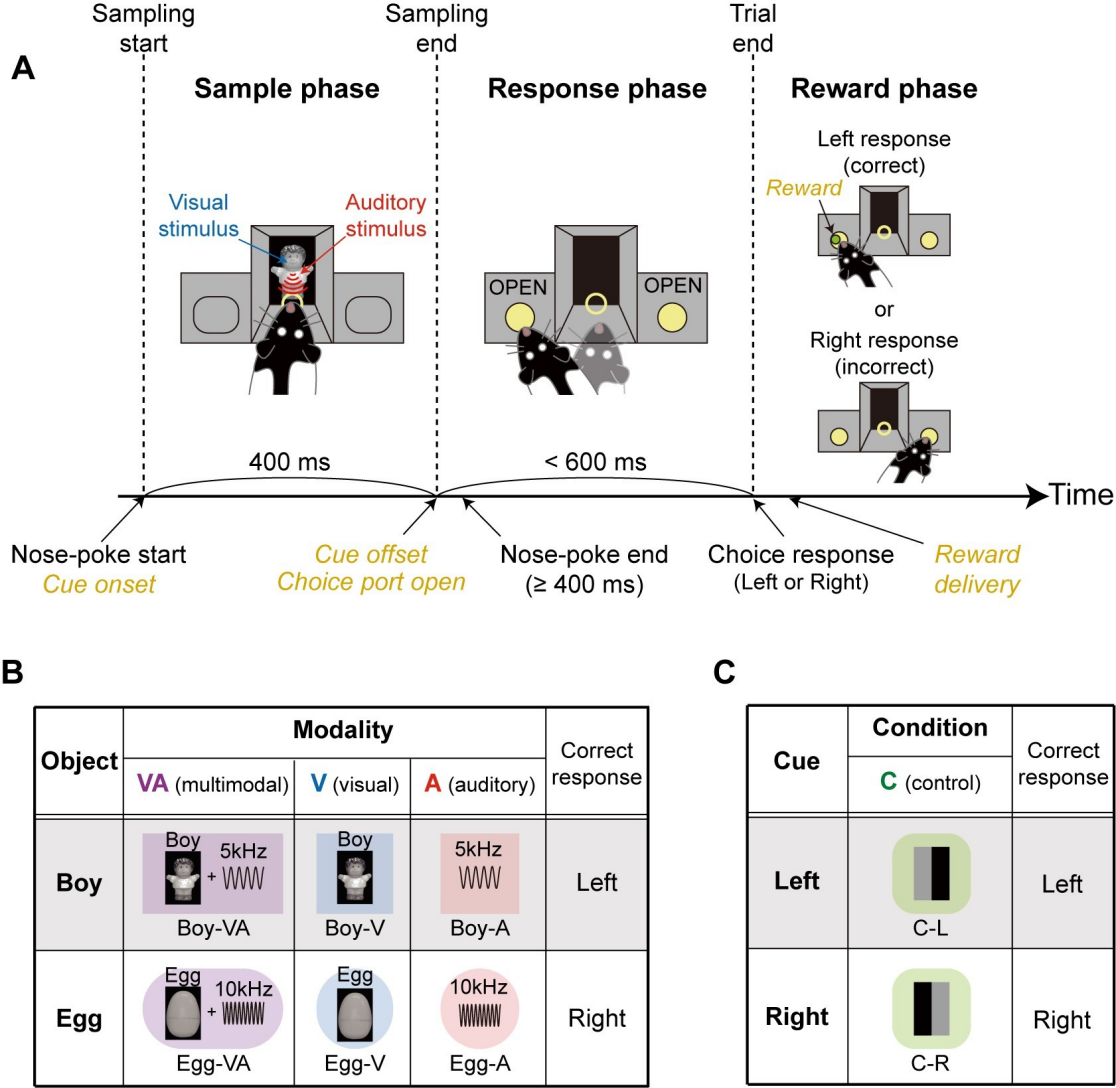
Multimodal object-recognition task. (**A**) Illustration of the apparatus and the trial structure of the multimodal object-recognition task. Rats sampled visual and auditory cues simultaneously or separately for 400 ms (sample phase) and then made a choice response based on the identity of the cue (response phase). A correct choice response resulted in a food reward (reward phase). (**B**) Object conditions used in the multimodal object-recognition task. Two different objects (*Boy* and *Egg*) were presented in 3 different modality conditions: multimodal (VA), visual (V), and auditory (A). The correct choice response was determined by the identity of the object. (**C**) Two simple visual cues were introduced as control (C) stimuli. Each control stimulus was also associated with either the left (C-L) or right (C-R) choice response (i.e., the same responses required by object conditions).

To test multimodal object recognition while controlling the sampling of the object’s unimodal (i.e., visual and auditory) attributes, we developed a behavioral paradigm for rats that would enable stable, simultaneous sampling of multimodal cues (**Fig 1A**; see **S1 Video**). The task tested object recognition by requiring the animals to identify a given object and produce a proper choice response associated with the object. The apparatus consisted of 3 ports: the center port was used to measure nose-poking by the rats to sample the cues, while the 2 side ports were used for obtaining rewards through choice responses. In the sample phase of this protocol, rats triggered the onset of an audiovisual cue (e.g., an image of a boy-shaped object with a 5 kHz sine-wave tone) by nose-poking the center hole and were required to maintain their nose-poke for at least 400 ms. This nose-poking behavior was trained during the shaping stage without a cue (see Methods for details). If a rat failed to maintain its nose-poke for 400 ms, the trial was stopped and the rat was allowed to retry the nose-poke after a 4-s interval (**S1 Fig**). After a successful (>400 ms) nose-poke, the cues disappeared, and doors covering left and right choice ports were opened simultaneously. In the response phase, rats were required to choose either the left or right port based on the sampled cue. In most trials, rats completed their choice responses within 600 ms (**S2A Fig**). A food reward was provided only after a correct choice response was made (reward phase), followed by a 2-s inter-trial interval.

To test the rat’s ability to recognize objects with multiple sensory modalities, we presented 2 different objects, *Boy* and *Egg*, consisting of different combinations of visual (images of a boy-shaped and an egg-shaped toy) and auditory (5 and 10 kHz sine-wave tones) attributes during the sample phase (**Fig 1B**). Objects were tested under 3 modality conditions: multimodal, visual, and auditory. In the multimodal condition, visual and auditory cues associated with an object were presented simultaneously during the sample phase. In unimodal—visual or auditory—conditions, only the object’s visual or auditory information was presented as a cueing stimulus. If the rat responded correctly to the object’s identity regardless of the modality condition, it was rewarded with a piece of cereal. The combination of audiovisual cues and stimulus-response contingency were counterbalanced across rats. In the control condition, rats learned to dissociate 2 simple visual stimuli composed of black and gray bars (**Fig 1C**). In this condition, the required left and right choice responses were the same as those in object conditions. The control condition was introduced primarily to exclude neurons that responded to a specific choice response in neural data analysis. In sum, 8 stimulus conditions were used in this task: 6 object conditions (2 objects × 3 modality conditions) and 2 control conditions.

### The PER is required for multimodal object recognition

**Fig 2:**
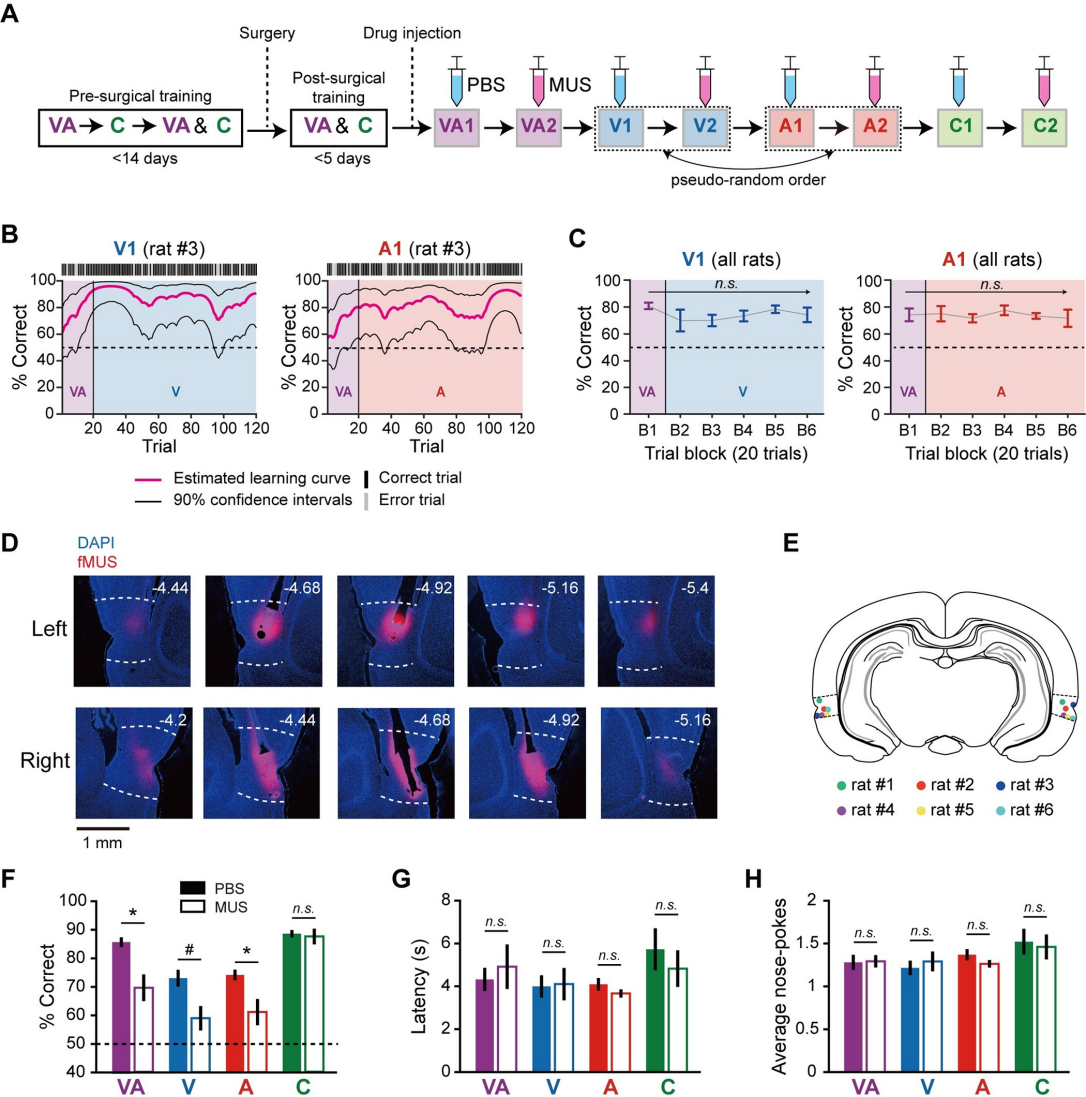
Necessity of the PER for multimodal object recognition. (**A**) Illustration of behavioral training and testing schedules for the PER-inactivation experiment. Note that animals were subjected to either the visual or auditory condition for the first time in PBS-injected visual (V1) or auditory (A1) sessions. (**B**) Estimated learning in V1 (left) and A1 (right) sessions of an example rat. In trial 21, where visual or auditory conditions were first introduced, the rat quickly adapted without additional learning. (**C**) On average, correctness did not significantly change across trials within the V1 (left) or A1 (right) session, indicating that rats could perform unimodal retrieval without additional learning. Each trial block consisted of 20 trials. (**D**) Histological verification of injection sites in the PER. White dotted lines indicate the border of the PER. The numbers on each section indicate the distance from bregma. (**E**) Summary of cannula-tip locations in all rats. (**F**) Behavioral performance in each condition was compared between PBS and MUS sessions. Performance was significantly impaired in all object conditions (VA, V, and A) by inactivation of the PER but remained intact in the control (C) condition. (**G**) The latency median did not change significantly after inactivating the PER. (**H**) The average number of nose-poke attempts did not change significantly after inactivating the PER. Data are presented as means ± SEM (*n* = 6; *p < 0.05, ^#^*p* = 0.062; n.s., not significant). Source data are available in **S1 Data**. MUS, muscimol; PBS, phosphate-buffered saline; PER, perirhinal cortex; SEM, standard error of the mean.

To test whether rats are able to perform the task when encountering the unimodal version of the multimodal condition for the first time following PER inactivation, we conducted a drug-inactivation experiment (*n* = 6). After training in multimodal and control conditions, rats were sequentially tested in separate sessions under multimodal, visual, auditory, and control conditions (**Fig 2A**). The order of visual and auditory sessions was counterbalanced across rats. For each condition, we first established baseline performance by injecting vehicle control (phosphate-buffered saline [PBS]) into the PER; we then tested performance in rats with an inactivated PER, achieved by injecting muscimol (MUS) bilaterally into the PER. Importantly, the sessions with PBS injections, either visual (V1) or auditory (A1) (**Fig 2A**), marked the first instances where rats were required to recognize objects originally learned multimodally, solely based on their unimodal sensory attributes. In a unimodal object recognition session, objects were presented multimodally (visual and auditory) for the first 20 trials. Subsequently, they were presented in a unimodal (visual or auditory) fashion for the remaining 100 trials, resulting in a total of 120 trials per session.

Performance dynamics of PBS-injected rats in visual and auditory sessions were displayed as learning curves, estimated from a given session (**Fig 2B**). Upon first encountering the visual or auditory condition (Trial 21), rats showed no significant drop in performance, and their performance remained stable until the end of the session. A statistical analysis of results for all PBS-injected rats revealed no significant increase or decrease in performance across all trial blocks (B1 to B6; 20 trials each) in either visual (F_(5,25)_ = 0.95, *p* = 0.47) or auditory (F_(5,25)_ = 0.22, *p* = 0.95; one-way repeated measures ANOVA) sessions (**Fig 2C**). These results indicate that rats easily recognized an object originally learned multimodally using one of its unimodal attributes, and this crossmodal recognition process required minimal training.

To verify the necessity of the PER in the task, we examined the effect of MUS injection on task performance. Histological results confirmed that MUS was successfully bilaterally injected into the PER (**Fig 2D** and **2E**). The average performance of rats (*n* = 6) in PBS sessions was significantly higher than predicted by chance (50%) in all conditions—multimodal (t_(5)_ = 21.2 *p* < 0.0001); visual (t_(5)_ = 7.8, *p* = 0.0005); auditory (t_(5)_ = 13.1, *p* < 0.0001); and control (t_(5)_ = 29.3, *p* < 0.0001)—as determined by one-sample *t* test. Inactivating the PER with MUS significantly decreased performance (F_(1,5)_ = 165.4, *p* = 0.0006; two-way repeated measures ANOVA) (**Fig 2F**). The interaction of drug and stimulus conditions was not significant (F_(3,5)_ = 1.99, *p* = 0.16, two-way repeated measures ANOVA). Further investigation into the effect of inactivation revealed performance deficits in multimodal (t_(5)_ = 3.72, *p* = 0.028), visual (t_(5)_ = 2.39, *p* = 0.062), and auditory (t_(5)_ = 3.45, *p* = 0.027) conditions (paired *t* test with Holm–Bonferroni correction), but not in the control condition (t_(5)_ = 0.37, *p* = 0.36; paired *t* test). Trial latency (i.e., from trial onset to end of choice) was not significantly affected by MUS injection (F_(1,5)_ = 0.13, *p* = 0.73; two-way repeated measures ANOVA) (**Fig 2G**). Nose-poking behavior was not affected by PER inactivation, as the average number of nose-poke attempts was not significantly different between PBS and MUS sessions (F_(1,5)_ = 0.92, *p* = 0.38, two-way repeated measures ANOVA) (**Fig 2H**). Collectively, these results demonstrate that the PER is necessary for object recognition in all modality conditions and that the decrease in performance is not attributable to deficits in general motor skills or to the loss of motivation.

### Object-selective neural activity in the PER is characterized by its transient and sequential firing patterns

**Fig 3:**
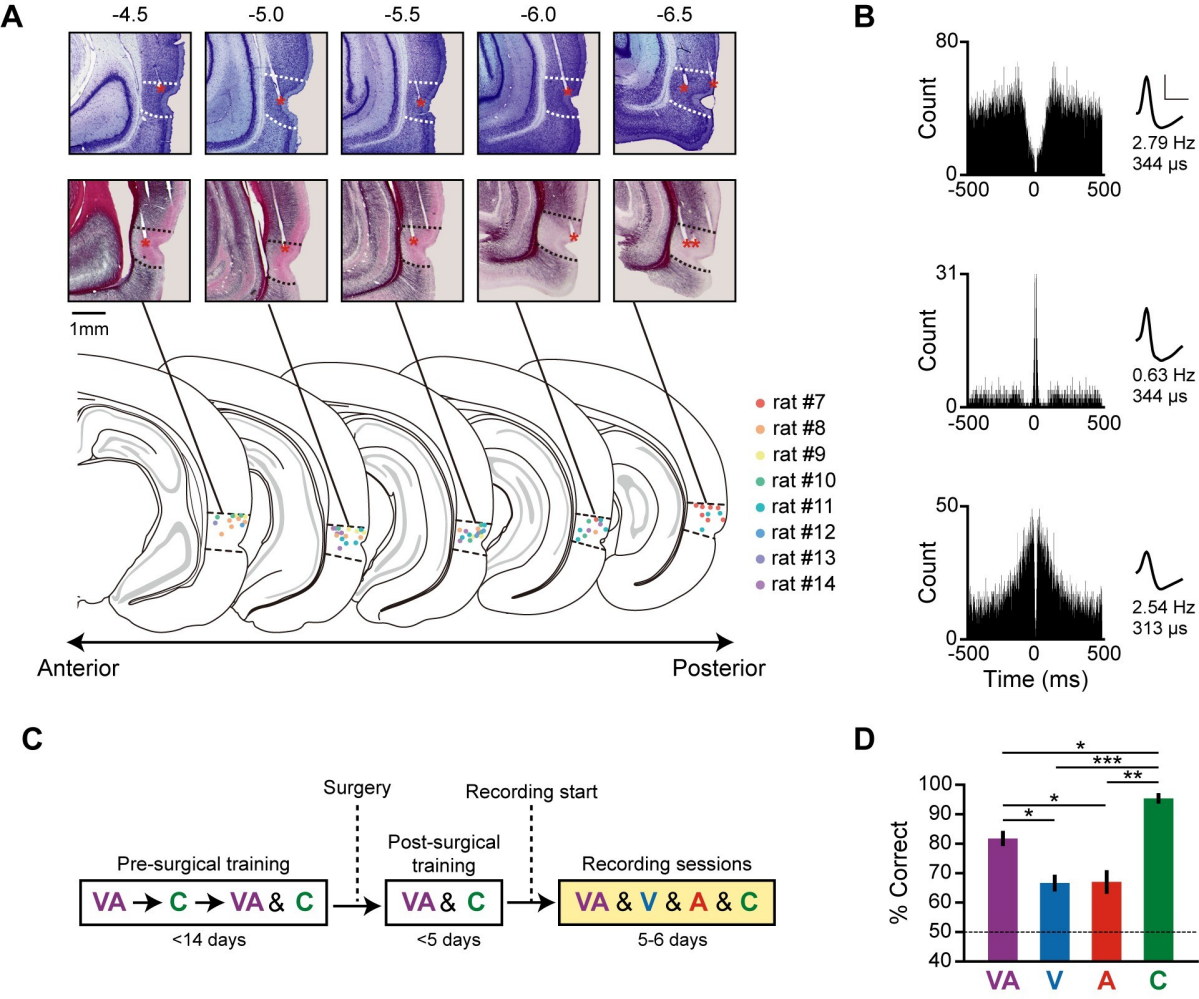
Single-neuron recordings during multimodal object recognition. (**A**) Histological verification of tetrode locations in the PER by Nissl (top) and myelin (middle) staining of sections across the anteroposterior axis. The estimated tetrode tip locations in all rats are summarized on the atlas (bottom). Dotted lines demarcate the borders of the PER. Tetrode tip locations are marked with red asterisks. The numbers above each section indicate the distance from bregma (mm). (**B**) Examples of single neurons classified according to their basic firing properties. Based on the autocorrelograms (left), cells were categorized as regular-spiking (top), bursting (middle), or unclassified (bottom). Scale bars in each spike waveform (right) indicate amplitude (vertical, 100 μV) and width (horizontal, 500 μs). The numbers below the waveform show the mean firing rate and spike width of each neuron. (**C**) Illustration of training and recording schedules for electrophysiological experiments. In the recording sessions, all stimulus conditions (VA, V, A, C) were pseudo-randomly presented within a session. Rats experienced visual or auditory conditions only in the recording sessions. (**D**) Behavioral performance in the first recording session. Although rats performed better in pre-trained multimodal and control conditions, they still showed better than chance-level performance in visual and auditory conditions. Data are presented as means ± SEM (*n* = 8; *p < 0.05, *p < 0.05, **p < 0.01, ***p < 0.001; n.s., not significant.). Source data are available in **S1 Data**. PER, perirhinal cortex; SEM, standard error of the mean.

Inactivation of the PER resulted in performance deficits whenever object recognition was required, regardless of the modality condition. To further understand the functions of the PER in multimodal object recognition, we searched for neural correlates of multimodal object recognition by recording single-unit spiking activity in the PER using tetrodes (**Fig 3A**). During each session, the rats performed a total of 180 to 250 trials, which took 60 to 100 min (see Methods for details). Based on their basic firing properties, most neurons could be classified into regular-spiking neurons (68%, 234 of 348), with bursting (24%, 82 of 348) and unclassified (9%, 32 of 348) neurons also being observed (**Fig 3B**), as previously reported [16,32]. In terms of anatomical subregions of the PER, the majority of the neurons were recorded within area 36 compared to area 35 (**S1 Table**).

Before obtaining single-unit recordings, rats were first trained in multimodal and control conditions; unimodal (visual or auditory) recognition conditions were introduced upon initiation of recordings (**Fig 3C**). All testing conditions (multimodal, visual, auditory, and control) were presented pseudo-randomly within a recording session. We confirmed that rats (*n* = 8) were able to successfully recognize objects in all conditions in their first recording session—multimodal (t_(7)_ = 12.36, *p* < 0.0001); visual (t_(7)_ = 5.88, *p* = 0.0006); auditory (t_(7)_ = 4.26, *p* = 0.0037); and control (t_(7)_ = 25.9, *p* < 0.0001)—as determined using one-sample *t* test (chance level, 50%) (**Fig 3D**). Significant differences in performance were also noted among conditions (F_(3,21)_ = 22.87, *p* < 0.0001, one-way repeated measures ANOVA), with rats performing significantly better in the multimodal condition than in either the visual (t_(7)_ = 3.43, *p* = 0.022) or auditory (t_(7)_ = 4.22, *p* = 0.016; paired *t* test with Holm–Bonferroni correction) condition. Performance in the control condition was significantly higher than that in all other conditions (control versus multimodal, t_(7)_ = 3.92, *p* = 0.017; control versus visual, t_(7)_ = 15.47, *p* < 0.0001; control versus auditory, t_(7)_ = 6.19, *p* = 0.0023; paired *t* test with Holm–Bonferroni correction). Similar behavioral results were observed in all recording sessions (**S2B Fig**). There was no evidence of new learning in either visual or auditory conditions even after repeating the recording sessions (**S2C Fig**).

**Fig 4:**
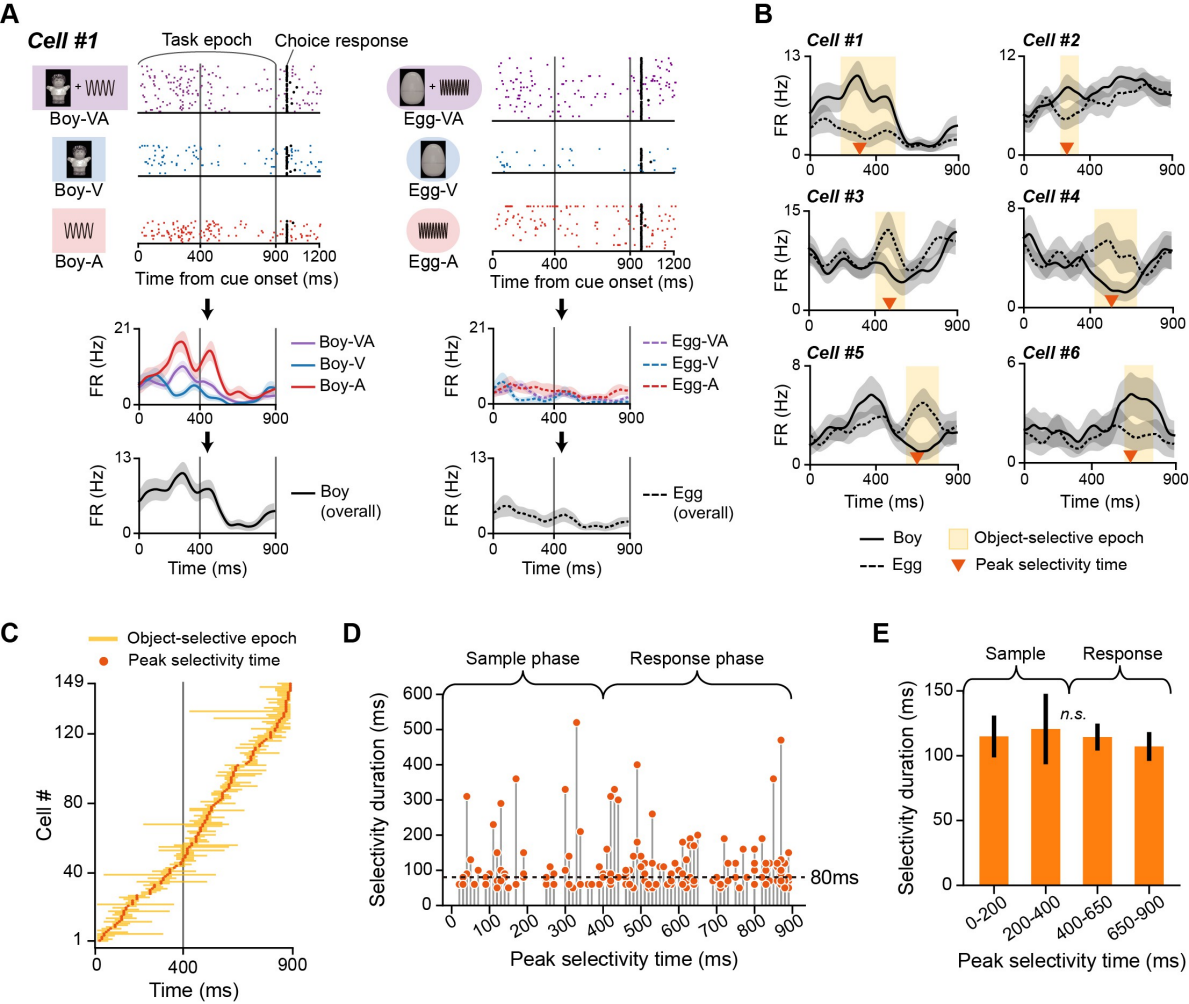
Object-selective firing patterns in the PER. (**A**) Raster plots (top) and spike density functions (bottom) of an example neuron for *Boy* (left) and *Egg* (right) object conditions. Overall firing rates for each object (black line) were obtained by averaging firing rates in different modality conditions (VA, V, and A). This sample neuron showed enhanced firing rates for the *Boy*, but not the *Egg* object (i.e., *Boy*-preferring neuron). Note that the interval from 0 to 900 ms after the cue onset, designated the task epoch, was the analysis target. (**B**) Example object cells in the PER showing selective firing patterns for an object over the object-selective epoch, indicated in yellow. Orange arrowheads indicate the peak selectivity time (i.e., time when selectivity was maximal). (**C**) Population object selectivity of all object cells and their peak selectivity times. The selective epoch of each object cell was marked and then aligned according to their peak selectivity time. The vertical gray line indicates the temporal boundary of the sample and response phases. (**D**) Peak selectivity time and duration of the selective epoch. Each dot indicates an individual object cell. The dotted line denotes the median selectivity duration (80 ms). (**H**) Comparison of selectivity durations between cells whose peak selectivity times appeared in different time ranges. No significant difference was found. Data are presented as means ± SEM (n.s., not significant). Source data are available in **S1 Data**. PER, perirhinal cortex; SEM, standard error of the mean.

We next sought to describe object selectivity of PER cells by determining how these neurons respond to different object identities regardless of sensory modality. To this end, we grouped all correct trials into different object and modality conditions and then calculated the firing rates associated with each condition during the task epoch, measured from the start of the sample phase to the end of the response phase (900-ms duration) (**Fig 4A**). Overall firing patterns were obtained by averaging firing rates in different modality conditions for each object, *Boy* and *Egg* (**Fig 4A** and **4B**, black lines). For each neuron, we defined an object-selective epoch as the period in which the firing rate for either object was significantly different from that of the other object in more than 5 consecutive time bins (10 ms/bin) (**Fig 4B**, example neurons #1–6).

It has been previously reported that PER neurons could fire for a specific behavioral response [16,30–32]. Therefore, the neural firing in the object-selective epoch could also be attributed to the choice response and not necessarily to the identity of the object, as each object was always associated with a specific choice response in our study. Hence, we aimed to exclude those response-selective cells further by examining the firing patterns in the control condition. Defining response-selective cells in relation to the control condition may not completely rule out the influence of the choice response factor. However, it allowed us to filter out those neurons that appeared to be purely associated with a specific choice response (**S4 Fig**). Those that were not purely tuned to a choice response were considered object-selective cells (hereafter, “object cells”). Selectivity was not maintained throughout the sample and response phases; thus, individual object cells were characterized by their transient firing patterns. Moreover, the time bin at which the firing rate difference between objects was maximal (i.e., peak selectivity time) occurred at various time points during the task epoch (**Fig 4B**).

To visualize the characteristics of object cells at the population level, we constructed a population object-selectivity plot (**Fig 4C**), in which object-selective epochs of individual object cells were marked and then aligned by their peak selectivity time. Interestingly, we observed a sequentially ordered firing of object-selective cells such that the population of object cells tiled the task epoch (from the sample phase to the response phase) with their object selectivity. We further investigated the possibility that object selectivity might be stronger in certain time bins, even when this sequential pattern was present. For this, we used the duration of selectivity as a measure of the magnitude of object selectivity and examined the relationship between the selectivity duration and peak selectivity time (**Fig 4D**). The median selectivity duration was 80 ms, confirming the transient nature of object-selective firing in the PER. We found no evidence that cells with greater selectivity were more active in certain time bins. Selectivity durations were not significantly different upon grouping cells into 4 temporal intervals based on their peak selectivity time (F_(3,145)_ = 0.14, *p* = 0.96; one-way ANOVA) (**Fig 4E**). Taken together, these observations indicate that object cells in the PER are characterized by their transient and sequential activity patterns, which tiled the entire task epoch. Notably, these characteristics were present regardless of whether the rats were sampling the cues (sample phase) or choosing a behavioral response in the absence of cues (response phase).

### Both visual and auditory information processing modes are found during object-selectivity firing in the PER

**Fig 5:**
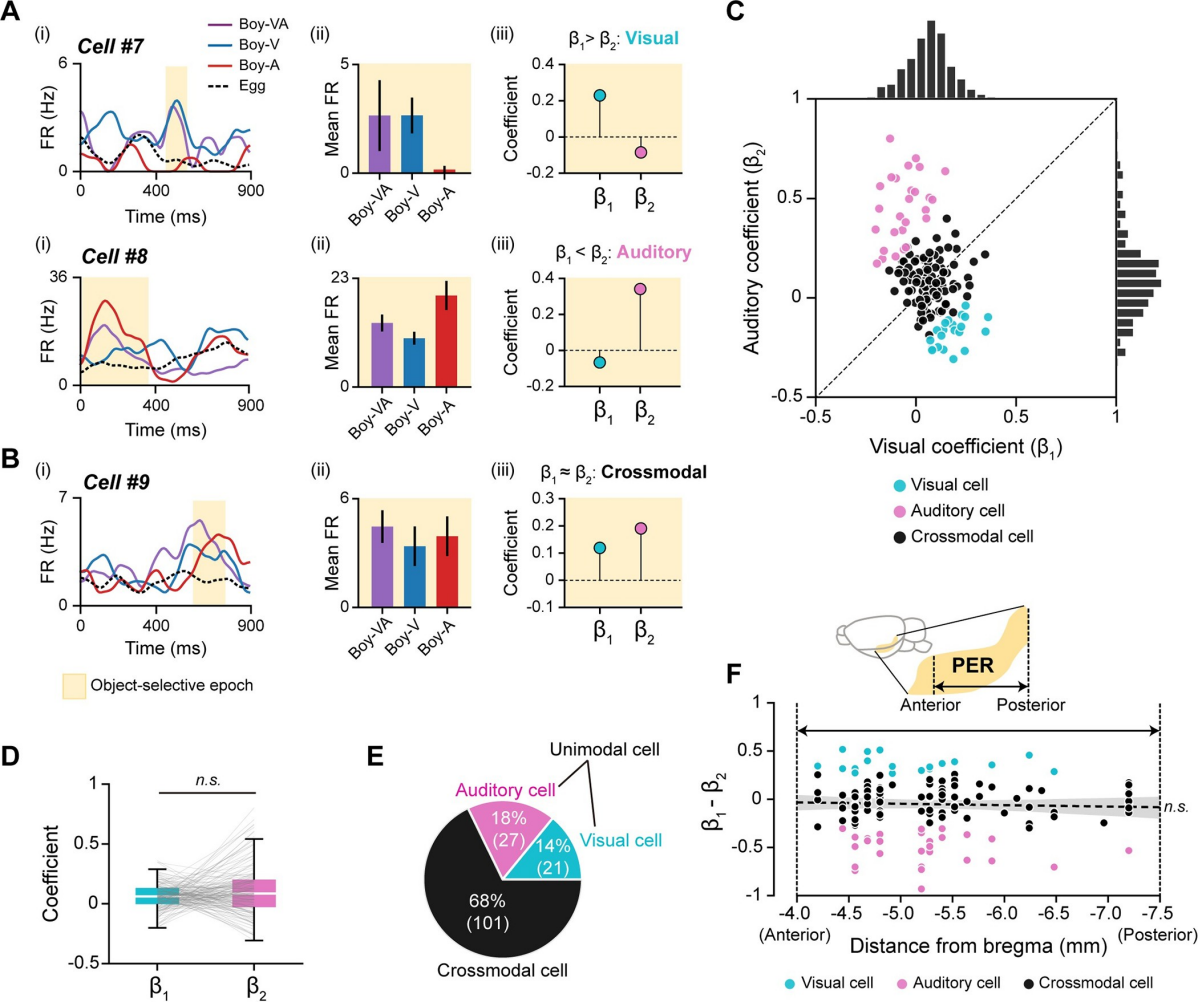
Unimodal and crossmodal response patterns of object cells in the PER. (**A**) Examples of unimodal cells that were responsive to either the visual or auditory attribute of an object during the selective epoch. Spike density functions (i) and mean firing rates within the object-selective epoch (ii). Multiple linear regression was applied to firing rates within the object-selective epoch to obtain β_1_ and β_2_—regression coefficients reflecting the magnitude of visual and auditory responses, respectively (iii). Cell #7 mainly responded to the visual attribute of *Boy* (β_1_ > β_2_), whereas cell #9 was responsive to the auditory attribute of *Boy* (β_1_ < β_2_). (**B**) Spike density functions (i), mean firing rates (ii), and regression coefficients (iii) of a crossmodal cell. The cell showed no specific bias for visual or auditory information processing, as indicated by similar β_1_ and β_2_ values. (**C**) Scatter plot and histograms of visual (β_1_) and auditory (β_2_) coefficients in all object cells. Neurons were classified as either visual (cyan) or auditory (pink) cells if the difference between visual and auditory coefficient was significant. Others were classified as crossmodal cells (gray). (**D**) Visual and auditory coefficients of all object-selective cells were not significantly different. Each line indicates an individual object cell. (**E**) Proportions of visual, auditory, and crossmodal neurons within the object cell category. Visual and auditory cells were grouped as a unimodal cell type. The numbers in parentheses denote the number of neurons. (**F**) Anatomical locations of object cells along the anteroposterior axis of the PER and their unimodal (or crossmodal) response patterns. Differences between β_1_ and β_2_ did not exhibit a significant linear relationship with the anatomical locations of the cells. The dotted black line indicates the linear regression line, and the shaded area is the 95% confidence interval (n.s., not significant). Source data are available in **S1 Data**. PER, perirhinal cortex.

If PER neurons solely focus on the identity of an object and its associated behavioral response, object-selective patterns should remain constant irrespective of the modality condition. Conversely, it could be argued that distinguishing between events associated with experiencing an object based on its distinct modality information is crucial for episodic memory. To determine whether PER object cells can encode a particular sensory modality, we applied multiple linear regression to firing rates during the object-selective epoch (see Methods for details). In this regression model, *β*_*1*_ and *β*_*2*_ are regression coefficients that represent the visual and auditory responsiveness, respectively, of the preferred object (i.e., the object condition with higher firing rates). Visual and auditory information-processing neurons within the PER were identified based on the relationship between *β*_*1*_ and *β*_*2*_ (**Fig 5A**). An example of an object cell that predominantly fired for the visual attribute of *Boy* is cell #7 (**Fig 5A-ii**), which had higher firing rates in multimodal and visual conditions compared with the auditory condition. This pattern is reflected in higher *β*_*1*_ versus *β*_*2*_ values (*p* < 0.05; two-sided permutation test) (**Fig 5A-iii**). Cell #8, on the other hand, was responsive to the auditory attribute of *Boy*, as its firing rates in the multimodal and visual condition were higher compared with those in the visual condition (**Fig 5A-ii**); it also had higher *β*_*2*_ than *β*_*1*_ values (*p* < 0.05; two-sided permutation test) (**Fig 5A-iii**). A crossmodal cell type, distinct from the unimodal cell type described above that exhibited no significant preference for a particular sensory modality, was also observed (**Fig 5B**). An example of a crossmodal cell is cell #9, which exhibited almost equal firing in response to both sensory modalities of its preferred object (*Boy*) (**Fig 5B-ii**); its *β*_*1*_ and *β*2 values were also similar (*p* ≥ 0.05; two-sided permutation test) (**Fig 5B-iii**).

To illustrate the patterns of modality correlates, we created a scatter plot of *β*_*1*_ and *β*_*2*_ values for all object cells (**Fig 5C**). We then verified that the PER system did not preferentially process one of the sensory modalities by first comparing *β*_*1*_ and *β*_*2*_ for all object cells (**Fig 5D**). This analysis showed no significant difference between *β*_*1*_ and *β*_*2*_ (W = 4794, *p* = 0.13; Wilcoxon signed-rank test), indicating that the PER did not have a significant bias toward a specific sensory modality. We then classified neurons based on the difference between their *β*_*1*_ and *β*_*2*_ values such that neurons whose *β*_*1*_ values were significantly higher than their *β*_*2*_ values were classified as visual cells, whereas those with significantly higher *β*_*2*_ than *β*_*1*_ values were classified as auditory cells (α = 0.05; two-sided permutation test). The remaining object cells were classified as crossmodal cells, exhibiting similar firing across both visual and auditory conditions. Although the majority of object cells were categorized as crossmodal (68%), both auditory cells (18%) and visual cells (14%) were identified (**Fig 5E**). The small difference in the proportion of visual and auditory cell categories was determined to be insignificant (χ^2^ = 0.89, *p* = 0.34; chi-square test). Detailed comparisons of selectivity patterns revealed that auditory cells exhibited stronger selectivity in the sample phase, and their selective period was longer than that of visual cells (U = 388.5, *p* = 0.03; Mann–Whitney U test) (**S5 Fig**). These findings suggest that modality information processing within the PER is heterogeneous, potentially enabling the retrieval of both object identity and its associated modality information.

Since the PER receives direct inputs from visual and auditory cortices [22,23], it is possible that the activity of visual and auditory cells in the PER is driven solely by inputs from the sensory cortices. If so, the posterior PER, where visual inputs are relatively dominant, might have more visual cells, whereas the anterior PER, which receives more auditory inputs, might possess more auditory cells. To test this hypothesis, we examined the relationship between the anatomical locations of cells along the anteroposterior axis of the PER and differences between visual (*β*_*1*_) and auditory (*β*_*2*_) coefficients (**Fig 5F**). We found no evidence for regional bias in coefficients in the posterior PER that would indicate the dominance of visual processing over auditory processing. Instead, visual and auditory cell types were evenly distributed along the anteroposterior axis of the PER. There was also no significant relationship between the anatomical locations and peak selectivity time of each neuron (**S7 Fig**). These results suggest that the activities of visual and auditory cells in the PER do not solely rely on inputs from visual and auditory cortices, respectively.

### Unimodal cells in the PER can further dissociate different modality conditions

If unimodal neurons are invariably activated by a specific sensory input, their activity levels should remain constant between multimodal and their preferred unimodal conditions, reflecting the fact that both conditions contain the same image or sound of an object. However, it is also possible that unimodal cells are further modulated by different modality conditions while maintaining their preferred visual or auditory information. To examine the modulation of firing rates across modality conditions, we defined a rate modulation index (RMI) based on Cohen’s *d*, where larger *d* values indicate a greater difference between groups (see Methods). RMIs, calculated as the difference in mean firing rates between modality conditions, were determined for multimodal and visual conditions (VA–V) and multimodal and auditory conditions (VA–A).

**Fig 6:**
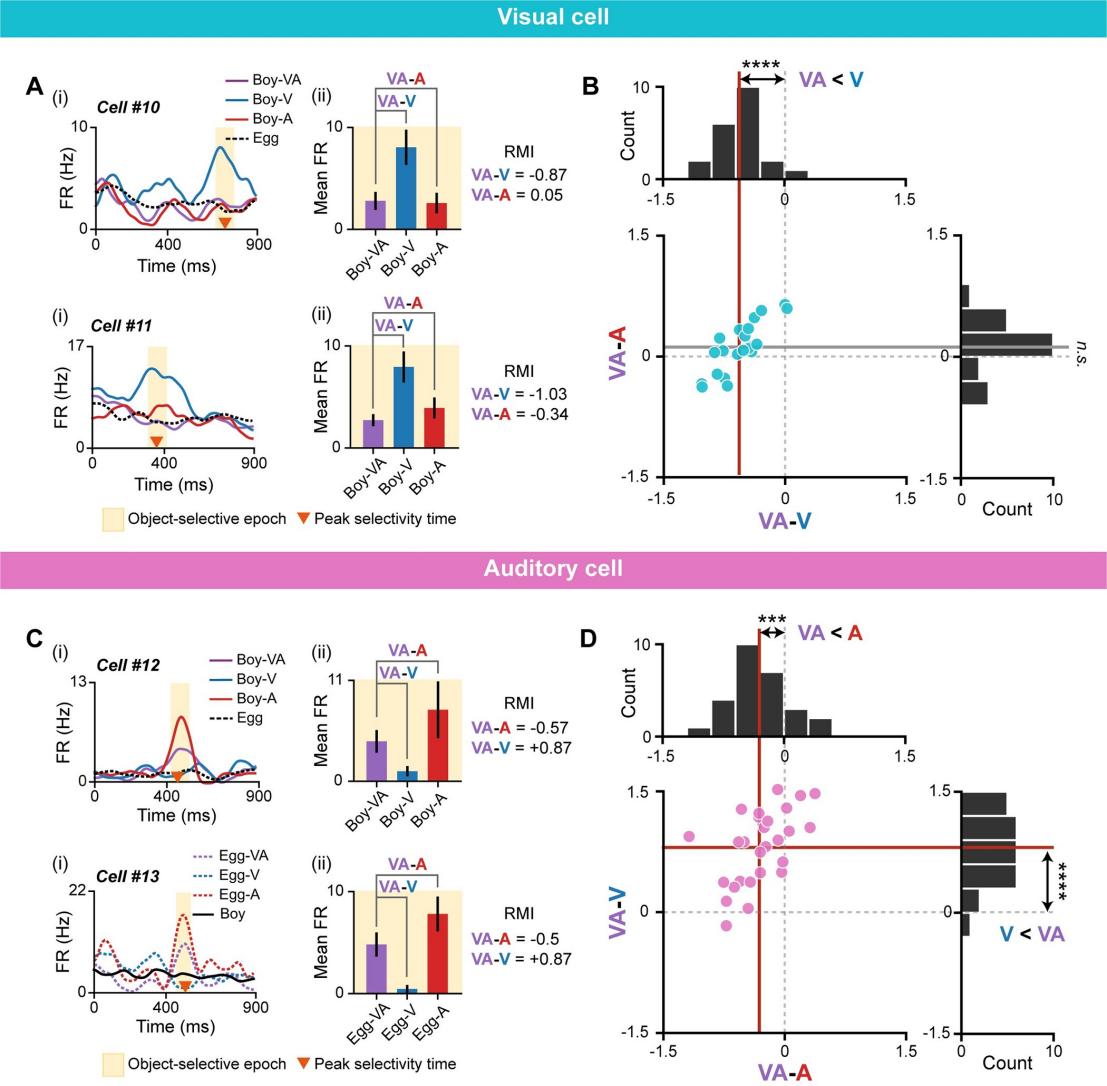
Further dissociation of modality conditions by visual and auditory cells. (**A**) Examples of visual cells (cells #10 and #11) demonstrating further dissociation of visual and multimodal conditions, but not multimodal and auditory conditions, as shown by their spike density functions (i) and mean firing rates within the selective epoch (ii). Differences in firing rate, quantified as RMI, showed that firing rates were different between visual and multimodal conditions (i.e., negative VA–V), but not between multimodal and auditory conditions (i.e., VA–A near zero). (**B**) Scatter plot and histograms of VA–V and VA–A in visual cells. For visual cells, the average VA–V (vertical red line) was significantly different from zero, whereas the average VA–A (horizontal gray line) was not. (**C**) Examples of auditory cells (cells #12 and #13) demonstrating further dissociation of all modality conditions, as shown by their spike density functions (i) and mean firing rates within the selective epoch (ii). RMIs showed that firing rates were different between auditory and multimodal conditions (i.e., negative VA–A), and also between multimodal and visual conditions (i.e., positive VA–V). (**D**) Scatter plot and histograms of VA–A and VA–V in auditory cells. The average VA–A (vertical red line) and average VA–V (horizontal red line) differed significantly from zero (***p < 0.001, ****p < 0.0001; n.s., not significant). RMI, rate modulation index.

Cells #10 and #11, examples of visual cells, are shown in **Fig 6A** with their RMIs. The subtracted value between multimodal and unimodal conditions (VA–V) was large and negative in both cells, indicating higher activities during the visual condition compared with the multimodal condition. Notably, visual cells exhibited “multisensory suppression,” such that firing rates were lower in the multimodal condition even though that condition contained the same visual information as the visual condition. However, VA–A values in both cells were small (near zero), indicating that their firing rates for multimodal conditions were not significantly different from those for auditory conditions. To visualize these patterns, we created scatter plots and histograms of RMI values for visual cells (**Fig 6B**). VA–V values for visual cells were significantly different from zero (t_(20)_ = 8.9, *p* < 0.0001; one-sample *t* test), indicating that visual cells further dissociated visual and multimodal conditions. However, VA–A values for visual cells were not significantly different from zero (t_(20)_ = 1.78, *p* = 0.091; one-sample *t* test), suggesting that visual cells are not a suitable neuronal substrate for dissociating multimodal and auditory conditions.

Next, we examined RMI values in auditory cells (**Fig 6C**). In cells #12 and #13, the RMIs between the multimodal and auditory conditions (VA–A) were negative, indicating relatively lower mean firing rates in the multimodal condition compared to the auditory condition, even though both conditions contained the same auditory information. That is, auditory cells, like visual cells, exhibited multisensory suppression. In addition, auditory cells further dissociated multimodal and visual conditions, showing relatively higher firing rates in the multimodal condition (i.e., positive VA–V). These patterns in auditory cells were visualized using scatter plots and histograms of RMI values (**Fig 6D**). Further analyses showed that VA–A values for auditory cells were significantly different from zero (t_(26)_ = 4.48, *p* = 0.00013; one-sample *t* test), indicating that these cells dissociated auditory and multimodal conditions. VA–V values for auditory cells were also significantly different from zero (t_(26)_ = 9.18, *p* < 0.0001; one-sample *t* test).

Collectively, these findings demonstrate that visual, auditory, and multimodal conditions can be distinguished based on the firing rates of single auditory cells, which exhibited a rank order of firing rate of A > VA > V. Further analyses revealed that crossmodal cells exhibited heterogeneous patterns of neural modulation compared with unimodal cells (**S8 Fig**). The multisensory suppression displayed by both visual and auditory cells could not be explained by familiarity-coding for the multimodal condition (i.e., repetition suppression; **S9 Fig**). Taken together, these results suggest that unimodal cell types in the PER do not merely respond to the presence or absence of specific modality information. Instead, they are capable of differentially representing different modality conditions by modulating their firing rates according to the specific modality conditions.

### The PER neuronal population can decode object identities in both a modality-specific and modality-invariant manner

**Fig 7:**
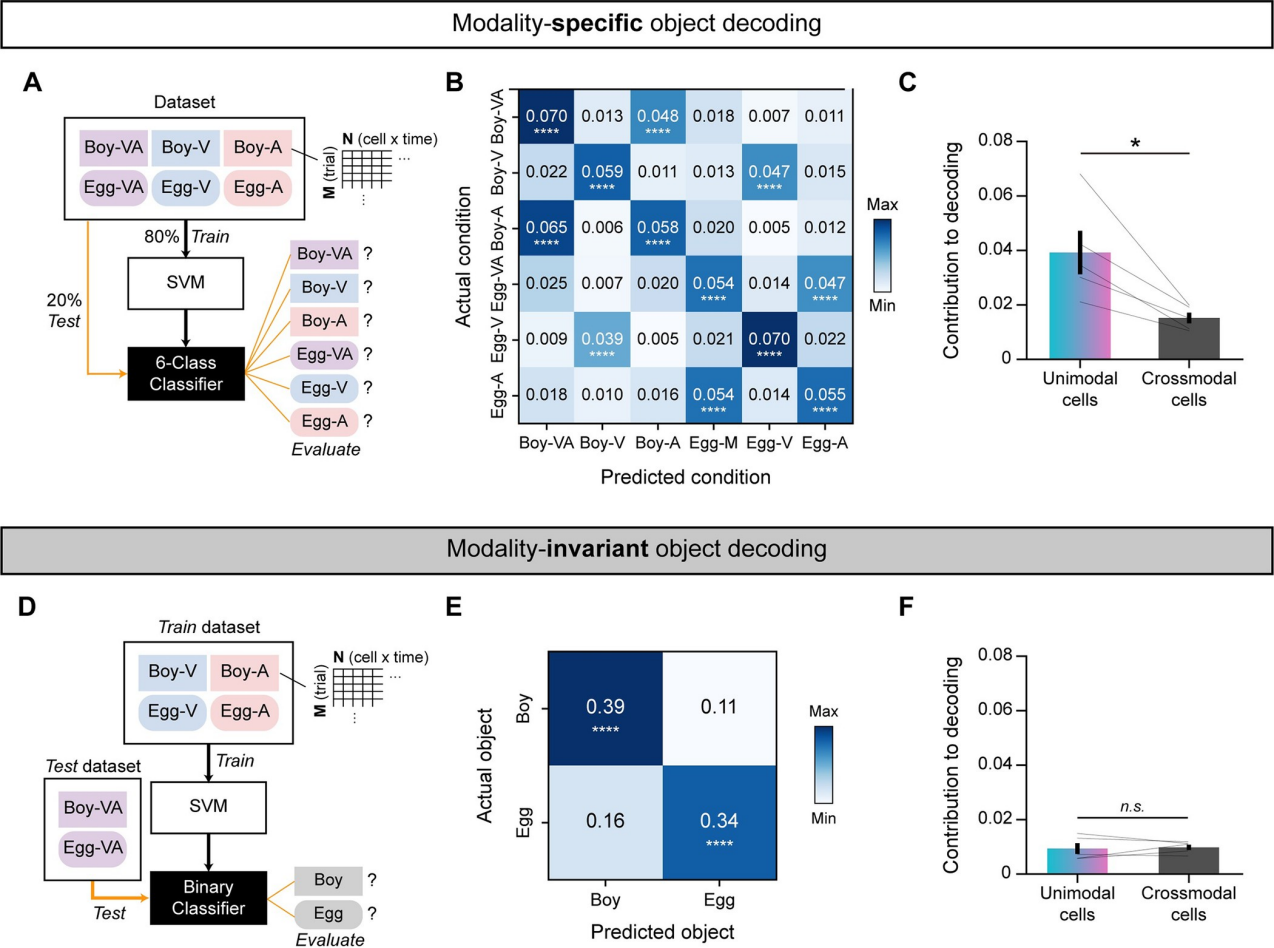
Modality-specific and modality-invariant decoding of object identities by the neuronal population in the PER. (**A**) Diagram summarizing modality-specific object decoding using a linear SVM. All 6 object conditions were utilized to train and test the SVM. (**B**) Confusion matrix showing the average decoding accuracy of the classifier depicted in **A** (*n* = 5). The population of PER neurons successfully decoded both object identities and modality conditions across all 6 object conditions, demonstrating modality-specific decoding. (**C**) Comparison of the contribution of a single neuron to decoding accuracy between unimodal and crossmodal cells showing a significantly higher contribution of unimodal neurons to this type of decoding (*n* = 5). (**D**) Diagram summarizing the decoding of multimodal trials based on unimodal information (i.e., modality-invariant object decoding) with a linear SVM. Note that the classifier was trained only for visual and auditory trials and tested only on multimodal trials. (**E**) Confusion matrix showing the average decoding accuracy of the classifier depicted in **D** (*n* = 5). The SVM trained with the unimodal trials successfully decoded multimodal trials, demonstrating the capability of modality-invariant decoding by the population of PER neurons. (**F**) The contribution of a single neuron to modality-invariant object decoding was similar between unimodal and crossmodal cells. Data are presented as means ± SEM (*p < 0.05, ****p < 0.0001; n.s., not significant). Source data are available in **S1 Data**. PER, perirhinal cortex; SEM, standard error of the mean; SVM, support vector machine.

Having described different categories of object cells and their heterogeneous activity patterns in response to objects with different sensory modalities, we next sought to assess how PER neurons support multimodal object recognition directly. To this end, we conducted a population-decoding analysis using 2 different linear support vector machine (SVM) classifiers to evaluate distinct multimodal object-recognition processes. These 2 classifiers were designed to test whether the PER neurons as a population could decode object identities in a modality-specific manner (classifier 1; **Fig 7A–7C**) or a modality-invariant manner (classifier 2; **Fig 7D–7F**). For each classifier, we sought to determine if decoding performance was significant and which cell categories contributed to the decoding.

For the first classifier, 6 object conditions—2 objects, each with 3 modality conditions—were decoded using a 6-class SVM classifier (**Fig 7A**). To create a dataset, we generated pseudo-populations of object cells and their firing rates during the task epoch for each rat (*n* = 5) by subsampling 5 trials from each condition (see Methods for details). We then employed a 5-fold cross-validation approach to train and test the dataset, repeating the procedures (subsampling, training, and testing) 100 times. A confusion matrix was created by averaging the proportions of actual and predicted conditions across rats (**Fig 7B**). In the confusion matrix, the proportion in the diagonal line (i.e., decoding accuracy) was significantly higher compared with that in the shuffled distribution (*p* < 0.0001), indicating the successful decoding of both object identities and modality conditions (permutation test). It should also be noted that there were significant instances where multimodal and auditory trials were cross-decoded (e.g., Boy-VA and Boy-A), or visual trials of different objects were cross-decoded (Boy-V and Egg-V), possibly reflecting qualitative differences between auditory and visual cells (**Figs 6** and **S5**).

Next, we sought to investigate how the activity patterns of crossmodal cells and unimodal cells (i.e., visual and auditory cells), identified in **Fig 5**, differently contributed to the population decoding results. We speculated that unimodal cells would make a greater contribution to the dissociation of modality conditions owing to their ability to dissociate not only visual and auditory inputs (**Fig 5C**) but also unimodal and multimodal conditions (**Fig 6B** and **6D**). For this analysis, we tested the respective contributions to decoding by quantifying the extent to which decoding accuracy decreased after shuffling data from a given cell category (see Methods for details). For example, to calculate the contribution of crossmodal cells to decoding, we shuffled trial labels (rows) only in features (columns) that were derived from crossmodal cells. We then assessed decoding accuracy before and after implementing this permutation, comparing the contribution of a single neuron in unimodal and crossmodal cell categories to decoding accuracy. This approach was selected because it maintains the number of input features despite the varying numbers of crossmodal and unimodal cells recorded in each rat (**S2 Table**). Single unimodal cells exhibited significantly higher contributions to decoding accuracy compared with individual crossmodal neurons (t_(4)_ = 3.7, *p* = 0.021; paired *t* test), indicating that the PER can decode modality-specific object information based on the activities of a limited number of unimodal cells (**Fig 7C**).

Next, we investigated whether the neuronal population in the PER could achieve modality-invariant decoding of object identities. Specifically, we sought to determine if object identities in multimodal trials could be decoded solely from unimodal trials by analogy to the ability of rats to identify objects learned multimodally based only on unimodal cues (**Figs 2B, 2C**, and **3D**). For this analysis, we trained the SVM to classify *Boy* and *Egg* objects using only unimodal trials (i.e., V and A). After training, we tested the classifier with multimodal trials to determine if the object identity could be successfully decoded (**Fig 7D**). The creation of pseudo-populations followed a process similar to that described in the previous section. In the confusion matrix, the proportion along the diagonal, indicating the accuracy of invariant object decoding, was significantly higher than that in the shuffled distribution (*p* < 0.0001; permutation test) (**Fig 7E**). Thus, successful modality-invariant decoding did not simply result from multimodal and unimodal conditions sharing the same choice response (**S10 Fig**). Decoding object identities of visual or auditory trials after training classifiers with multimodal trials, which is the opposite of **Fig 7D**, also revealed significant decoding accuracy in both visual and auditory conditions (**S11 Fig**). Collectively, these results indicate that the population of PER neurons could achieve modality-invariant decoding of object identities.

Finally, we examined how different cell categories contributed to invariant object decoding (**Fig 7F**). To measure the contribution to decoding, we quantified the degree of decrease in decoding accuracy after shuffling data from a given cell category (i.e., unimodal or crossmodal), as in **Fig 7C**. In contrast to the differentiation of modality information, the contribution of a single neuron to decoding performance was minimal for invariant objects. In addition, both crossmodal and unimodal cells contributed similarly to decoding (t_(4)_ = 0.29, *p* = 0.78; paired *t* test) (**Fig 7F**). These results suggest that the PER can also accomplish modality-invariant recognition of objects and further that this process is supported by population activity patterns of multiple neurons rather than by a limited subset of single neurons.

## Discussion

In the current study, we investigated how the PER contributes to multimodal object recognition using a behavioral paradigm in which rats were required to identify a given object regardless of its modality information to produce a correct choice response associated with the object. Rats identified the objects correctly even when provided only a unimodal feature, and the PER was required for normal performance. Single-unit recordings revealed that PER neurons exhibited transient object-selective signals that fired sequentially throughout the entire task epoch. Certain object-selective neurons responded primarily to visual or auditory attributes of an object (unimodal cells), whereas others exhibited equivalent selectivity across different object modalities (crossmodal cells). Unimodal cells further dissociated different modality conditions through modulation of their firing rates. Lastly, using a population-decoding analysis, we found that the PER was capable of accomplishing both modality-specific and modality-invariant object recognition. Specifically, modality-specific decoding was enabled by a small number of unimodal cells, whereas modality-invariant decoding was achieved through collective activity patterns of a relatively large number of neurons, regardless of their cell types (i.e., unimodal or crossmodal cells). Overall, our findings suggest that the PER supports multimodal object recognition by engaging in both modality-invariant recognition of objects and separation of object experiences based on modality information.

### Necessity of the PER in processing the combination of multimodal features

As previously reported, PER inactivation in our study resulted in performance deficits in the multimodal object-recognition task [5,7]. However, in the control condition, where simple visual stimuli with low feature complexities were presented, inactivating the PER did not result in a significant performance deficit. These findings align with previous literature that the PER primarily processes stimuli with high feature complexity, which are formed by combinations of multiple low-level features [33,34]. While this hierarchical processing has typically been discussed within the context of the visual modality [12,35,36], our results suggest the possibility that such hierarchical processing may also occur in multimodal situations. It is noteworthy that despite the seemingly low feature complexity of the auditory tones used in the multimodal condition, inactivating the PER led to performance deficits when rats first experienced auditory-only situations, unlike the control condition. This result suggests that the auditory features were combined with visual features to create a high-level feature representation of an object, leading to the involvement of the PER even during the processing of simple auditory stimuli.

While behavioral results strongly suggest the involvement of the PER in multimodal object recognition, it remains uncertain whether the PER is especially important in the integration of multimodal features. For example, performance deficits observed across all modality conditions (i.e., multimodal, visual, and auditory conditions) could occur even if the PER processes visual and auditory information separately [6]. Indeed, it has been reported that the rodent PER is engaged in various tasks that employ visual- or auditory-only cues [37,38]. A similar issue is applicable to previous behavioral experiments that reported performance deficits in tests of spontaneous object recognition in both crossmodal and unimodal conditions [5–7]. Therefore, this highlights the importance of concurrently examining both behavioral and physiological outcomes within a study to fully understand the integration and separation of unimodal features during multimodal object recognition.

### Possible advantages of transient and sequential object selectivity within the PER

Since we controlled the sampling and response times of rats precisely by compelling nose-poke behaviors, we were able to describe the detailed temporal dynamics of neuronal activity during multimodal object recognition. We discovered that individual PER neurons elicited transient object selectivity, which spanned not only the sample phase but also the response phase. While we attempted to mitigate the influence of the choice response factor by analyzing the control condition (**S4 Fig**), it is possible the object selectivity measured in the response phase might still be affected by choice responses. However, recent studies on working memory, especially the ones emphasizing more dynamic processes within and across task phases, have shown that firing patterns cannot be distinguished clearly by artificially defined task phases [39,40]. For example, it has been reported that it is difficult to clearly separate the “sample phase” neuron from the “delay phase” neuron. Rather, different neurons showed heterogeneous firing patterns even within a task phase, leading to sequential and continuous firing patterns across multiple task phases at the population level [41,42]. Additionally, our previous study has shown that the PER neurons can represent information seemingly unrelated to the demand of the current task phase [16]. Specifically, a significantly larger proportion of neurons represented the interaction of the object and choice response in the post-choice phase compared to the pre-choice phase, despite no task-related cue available in the post-choice phase. Taken together, these results suggest that the task phases operationally defined by experimenters may not always serve as the clear boundaries for the neural activity patterns. For these reasons, we did not restrict the analytic window to a single task phase (e.g., sample phase) in the current study, mainly to discover sequential neural dynamics that spanned across sample and response phases.

Although the sequential nature observed across the sample and response phases has rarely been observed in the PER, it is commonly reported in other brain regions, such as the prefrontal cortex [42], posterior parietal cortex [43], and hippocampus [44]. Sequential representations can function as mnemonic units, as evidenced by the sequential reactivation of place cells during hippocampal sharp wave ripples [44,45]. Therefore, the sequential coding in the PER may imply that the region integrates the sample and response phases into a unified event. Meanwhile, sequential coding itself has been reported to be beneficial for various aspects of memory processing. That is, a sequential activity pattern is a way to achieve high-dimensional information processing, which can enhance memory capacity and mitigate memory loss [46]. It has also been suggested that sequential firing patterns within the medial temporal lobe represent temporal information of events, as exemplified by time cells in the hippocampus [47]. The lateral entorhinal cortex, which receives extensive monosynaptic inputs from the PER, has also been reported to represent task-related time information [48]. The PER itself is also known to process time information in association with object information, although the proportion of time-coding neurons is smaller compared to the hippocampus [49]. These findings collectively suggest the PER’s potential contribution to remembering temporal information, which may be utilized as the “when” component of episodic memory, presumably in the hippocampus.

### Potential roles of the PER single neurons in both integrated and segregated encoding of multimodal object information

Previous studies have described the PER as an associative area in terms of both its physiological characteristics [29] and task-related firing patterns [26,50]. For example, neurons in the PER are known to be responsive to associations not only between 2 paired visual stimuli [29] but also between stimuli and their locations [50], demonstrating the PER’s associative properties in various domains. The PER was also theorized to primarily function in the “unitization” process [51]. That is, it was suggested that the PER plays a role in situations where complex features of a single entity must be integrated, such as when experiencing a complex object with multisensory information rather than sampling a simple cue. Based on these hypotheses, the PER is expected to encode multimodal objects in an integrated fashion instead of representing information of a single object separately based on its modality. Consistent with these expectations, we discovered that most object cells in the PER exhibit constant selectivity patterns, irrespective of the modality condition (i.e., crossmodal cells). We believe that our task requirements were suitable for facilitating the unitization process, as the multisensory cues were spatially and temporally congruent, and each audiovisual combination required the same behavioral response. Thus, our results provide experimental support for the idea that single neurons in the PER can encode multimodal objects in a unitized representation. Additionally, it is worth mentioning that most of the PER neurons were recorded in area 36, compared to area 35, where more intrinsic connections potentially render stronger unitized representations [23,52,53]. If more recordings had been conducted in area 35, we might have observed a higher proportion of crossmodal cells and their stronger modality-invariance within the PER, compared to the current findings.

It should also be noted that a significant proportion of unimodal cells in the PER primarily responded to a specific sensory modality when processing object information, an outcome that is not expected based on previous literature reports [31,51]. These neurons not only preferred a particular sensory modality, they also further dissociated unimodal and multimodal conditions through modulation of their firing rates. These unimodal activities could be interpreted as purely perceptual signals that reflect the physical attributes of visual and auditory cues. The perceptual-mnemonic hypothesis, which posits that the PER is involved in both perception and memory, may further support the interpretation that unimodal cells indeed represent perceptual information [34,54–57]. However, it is unlikely that unimodal neurons simply mirrored low-level perceptual features of the stimuli. If unimodal cells represented perceptual signals originating from the visual (or auditory) cortex, it is likely that the posterior (or anterior) PER would have more visual (or auditory) cells since visual (or auditory) input is more dominant in the corresponding area. Instead, we observed that each cell category appeared to be equally distributed along the anteroposterior axis of the PER. Moreover, unimodal cells showed modulation by their non-preferred sensory modality, indicating that they were not simply responding to the presence of a specific modality cue. Thus, unimodal cell activity in this area could have been driven by intrinsic connections within the PER [29] or by inputs from other higher-order associative areas, such as the prefrontal cortex and hippocampus [58–60]. Given that the PER is part of the medial temporal lobe memory system, it can be argued that unimodal representations exist for memory encoding and retrieval rather than for simple sensory processing. To determine whether these firing patterns indeed reflect memory processes, future studies are needed to compare activity during incorrect trials or investigate the changes of firing patterns during the learning process.

Although our behavioral and physiological data suggest that the PER is involved in processing both visual and auditory features, the region did not seem to treat visual and auditory features uniformly. Visual cells and auditory cells exhibited different characteristics in various aspects, even though the correctness for each modality was comparable. Specifically, visual cells tended to exhibit weaker (i.e., shorter) object selectivity (**S5 Fig**), which was reflected in poorer decoding of object identities in visual trials compared to auditory trials (**Fig 7B**). In terms of modulations to the multimodal condition, auditory cells discriminated the unimodal and multimodal conditions less evidently compared to visual cells (**Fig 6**), leading to poorer dissociations of the multimodal and auditory conditions in population decoding (**Fig 7B**). These results collectively reveal that visual and auditory information is processed differently within the PER. However, different experimental conditions may yield different results. For example, patterns of visual and auditory information processing may differ when auditory features are made to have psychophysically equivalent complexity to visual ones [61]. Further studies are needed to fully elucidate the exact coding scheme for processing different modalities.

### Dual functions of the PER in multimodal object recognition: Invariant recognition and episodic memory

From a computational standpoint, an object-recognition system is expected to be able to recognize an object through an invariant representation, even if the object’s physical attributes (e.g., size, position, and view) are modified [12]. In multimodal object recognition, it is also important that objects be identified as invariant of modality information. This modality invariance can be attained by individual neurons, as exemplified by “concept cells” that fire invariantly to both the image and voice of a person [62–64]. Crossmodal cells in our study shared some commonalities with concept cells from the human hippocampus as they showed some degree of invariance to modality information when coding object identities. However, we discovered that individual crossmodal cells within the PER do not contribute significantly to modality-invariant object recognition, making contributions to decoding accuracy similar to those of the unimodal cell type. This may be because crossmodal cells were not fully invariant to modality conditions but instead showed slight modulations in response to different modality conditions of objects (**S8 Fig**). More detailed investigations of concept-like representations also suggest that firing patterns of individual neurons can be heterogeneous and that population-level activities are better suited to achieve invariance [65,66].

In addition to the invariant recognition process, we discovered that populations of PER neurons can perform modality-specific object decoding, a process that seems to be counterproductive for the invariant identification of objects. However, in terms of episodic memory, segregation of similar events (i.e., pattern separation) is a crucial computational step for encoding and retrieving correct memory [33,67]. In cases where a single object is experienced by multiple senses, each experience should be separated into different episodes, even though they involve the same object. Pattern separation for episodic memory is thought to be primarily implemented in the dentate gyrus [68,69]. However, since a significant portion of information received by the dentate gyrus relies on connections between the PER and entorhinal cortex, modality-specific information in the PER could be an essential source for pattern separation within the dentate gyrus. In addition, it has been suggested that the PER itself can support pattern separation when 2 visual stimuli are highly overlapped as they morph into each other [57]. Validating the relationship between modality-specific representations and pattern separation will require future studies that systematically manipulate the amount of information from each modality.

## Methods

### Subjects

Male Long–Evans rats (10 week old; *n* = 14) were obtained and individually housed in a temperature- and humidity-controlled animal colony. Rats were allowed free access to food and water for 1 week before food restriction, during which they were allowed only 2 to 3 pellets (6 to 10 g) per day to maintain them at approximately 80% of their free-feeding body weight (approximately 400 to 420 g). Rats were housed on a 12-h light/dark cycle (lights on at 8 AM), and all experiments were performed in the light phase. All animal procedures were performed in accordance with the regulations of the Institutional Animal Care and Use Committee (IACUC) of Seoul National University (SNU-200504-3-1).

### Behavioral apparatus

The apparatus consisted of an elevated chamber (22 × 35 × 40 cm; 94 cm above the floor) with a custom-built device (22 × 18 cm) at the front of the chamber that was used for manipulating cues and measuring animal behaviors with Arduino MEGA (Arduino) and MATLAB (MathWorks). The frame of the device was printed with a 3D printer (Mojo; Stratasys), and the center of the device contained a transparent acrylic window (8 × 10 cm) with a nose-poke hole (diameter, 2.4 cm; depth, 1.5 cm). The hole was equipped with an infrared sensor to measure the onset and maintenance of nose-poking behaviors during cue sampling. An LCD panel (3.5 inch; Nextion) for presenting a visual cue, operated by Arduino, was positioned behind the acrylic window. Directly behind the LCD panel was a 3W speaker, operated through an Arduino music player module (DFPlayer Mini Mp3 Player; DFRobot), for presenting an auditory cue. The device contained 2 identical ports located on the left and right sides. Each port was equipped with a servo-motorized door for controlling access and infrared sensors for detecting choice responses. Another servo-motorized door located on the top of the port controlled the gravity-fed delivery of a pre-loaded food reward to the choice port. A small buzzer was placed on the back of the chamber to provide auditory feedback about the correctness of the rat’s choice. The experimental room was dimly lit with a circular array of LEDs (0.8 lux), and white noise (68 dB) was played through loudspeakers to block out uncontrolled noise.

### Behavioral paradigm

#### Shaping

After 6 days of handling, a shaping stage was conducted during which rats learned how to maintain nose-poking of the center hole. The required duration for nose-poke was 10 ms beginning in the first shaping trial and then was increased by 10 ms for each successful poke to a maximum of 400 ms. When rats failed to maintain their nose-poke for the required duration, the trial was stopped, and a 4-s interval was given together with auditory feedback (buzzer, 230 Hz, 76 dB). Once rats successfully completed 100 trials of 400-ms nose-pokes within a 30-min session, they advanced to the multimodal object-recognition task. All rats completed their shaping in 3 to 5 days.

#### Multimodal object recognition–training

Rats learned to make an associated choice response based on a presented cue. Initially, the rats were trained under multimodal object conditions (designated VA), in which a combination of visual and auditory cues was presented simultaneously. The visual cues used were 2D photographic images of 2 junk objects—a boy and an egg—presented via an LCD panel (1.6 lux). The 2 object images were adjusted to equal luminance by matching their average gray values in Photoshop (Adobe). Auditory cues were 5 kHz and 10 kHz sine-wave tones (81 dB) that were briefly repeated twice (on-time of 180 ms and off-time of 20 ms). Each object was associated with either a left- or right-choice response. The combination of audiovisual cue and stimulus-response contingency was counterbalanced across rats. An object containing a boy (or egg) image was called a *Boy* (or *Egg*) object, regardless of the auditory cue associated with it. Nose-poking to the center hole simultaneously triggered the onset of visual and auditory cues, which remained present for up to 400 ms while the rat maintained the nose-poke. If rats failed to maintain the nose-poke for at least 400 ms (i.e., prematurely withdrawn nose-poke), cues disappeared, and the auditory feedback was given together with a 4-s interval. A pseudo-random stimulus was presented on the next nose-poking regardless of the previously experienced stimulus. Prematurely withdrawn nose-pokes did not increase trial numbers. In successful nose-pokes (>400 ms), the doors covering the left and right choice ports were opened, allowing the rat to access one of the choice ports. A correct choice response resulted in the delivery of a food reward, whereas incorrect responses resulted in auditory feedback without a food reward together with an 8-s inter-trial interval. Rats performed 100 to 120 trials in total within a session. After rats exceeded the learning criterion (>75% correct in all conditions for 2 consecutive days), they learned the same task but using 2 simple visual cues as a control (C) condition. Rats that exceeded the learning criterion in the control condition were then trained with both multimodal objects and control stimuli within a session until they reached the criterion. After completing all training procedures, rats underwent either cannula or hyperdrive implantation surgery (see below for details). After surgery, they were again trained simultaneously on multimodal and control conditions and then proceeded to the test phase.

#### Multimodal object recognition–testing

Unimodal conditions (visual or auditory) were introduced for the first time in the test phase of multimodal object recognition. In the visual (V) condition, only the boy or egg image was presented without an auditory cue. In the auditory (A) condition, only a 5- or 10-kHz sound was presented without an image. Rats were required to make the same choice response associated with the multimodal object based on the unimodal stimulus. In the drug-infusion study, rats were serially tested under multimodal, visual, auditory, and control conditions in separate sessions, and 120 trials were performed per session. In the electrophysiological study, all 8 conditions (2 objects × 3 modality conditions plus 2 control stimuli) were pseudo-randomly presented within a session, and rats performed 180 to 240 trials per session (see below for details). Each session for both drug infusion and electrophysiological experiments was conducted on a separate day.

### Drug infusion

The guide cannula (24 gauge, 18 mm long), internal cannula (30 gauge, 19 mm long), and dummy cannula (30 gauge, 19 mm long) were built in-house. A surgery targeting the PER bilaterally was performed by first carefully retracting the left and right temporalis muscle, after which 2 holes were drilled bilaterally on the skull surface (4.8 mm posterior to bregma, 5.2 mm lateral to the midline). Guide cannulas were angled 15 degrees outward, lowered to 7 mm below the cortical surface, and chronically fixed with 4 anchoring screws and dental cement. The procedure was completed by placing dummy cannulas inside the guide cannulas. During insertion, the tips of the internal and dummy cannulas protruded 1 mm from the tip of the guide cannulas. Cannulas were cleaned at least once every 2 d. The drug infusion schedule was started after all rats had been retrained to multimodal and control conditions. PBS (0.5 μl per site) and the GABA-A receptor antagonist, muscimol (MUS; 0.5 μl per site), were bilaterally injected into the PER on alternate days using a Hamilton syringe (10 μl). After 1 rat (rat #5) showed immobilization side effects following muscimol injection, the injection amount was reduced to 0.3 μl. Drug infusions were made 20 min before the start of the behavioral experiment. Rats were tested in each condition on a different day in the following order: multimodal, unimodal (visual and auditory), and control. The order of visual and auditory sessions was pseudo-randomized for each rat. At the end of the experiment (20 min before sacrifice), the diffusion range of MUS was estimated by injecting rats with fluorescent BODIPY TMR-X–labeled MUS (fMUS) and monitoring fMUS by fluorescence microscopy.

### Hyperdrive implantation

The hyperdrive containing 27 tetrodes was built in-house. Tetrodes were prepared by winding together 4 formvar-insulated nichrome wires (diameter, 17.8 μm) and bonding them with heat. Impedance was reduced to approximately 200 kΩ at 1 kHz by gold-plating wires using a Nano-Z plating system (Neuralynx). For targeting the PER along the anteroposterior axis, a 12G stainless-steel cannula bundle housing 27 tetrodes was formed into an elliptical shape (major axis, 3.4 to 3.8 mm; minor axis, 2 to 2.4 mm). After performing surgery to target the right hemisphere of the PER, as described above, a hole sized to fit the tetrode bundle was drilled on the skull surface. The bundle tip was angled 12 degrees outward and lowered until it touched the cortical surface, after which the hyperdrive was chronically fixed with 11 anchoring screws and bone cement.

### Electrophysiological recording

After allowing 3 d to recover from surgery, rats were reacclimated to experimentation by handling for 4 d and then retrained to perform the multimodal object recognition task under multimodal and control conditions. Individual tetrodes were lowered daily. After most of the tetrodes had reached the PER and rats showed greater than 75% correct responses in both multimodal and control conditions for 2 consecutive days, recording sessions were begun. In the recording sessions, the unimodal condition was introduced for the first time, such that multimodal, visual, auditory, and control conditions were all presented pseudo-randomly during a session. Recordings were conducted in each rat for 5 to 6 d, and no attempt was made to record the same neuron across days. Neural signals were amplified 1,000 to 10,000-fold and bandpass filtered (300 to 6,000 Hz) using a Digital Lynx data-acquisition system (Neuralynx). Spike waveforms exceeding a preset threshold (adjusted within the range of 40 to 150 μV) were digitized at 32 kHz and timestamped.

### Histology

Rats were killed with an overdose of CO_2_ and transcardially perfused first with PBS and then with a 4% (v/v) formaldehyde solution. The brain was extracted and maintained in a 4% (v/v) formaldehyde-30% sucrose solution at 4°C until it sank to the bottom of the container. The brain was subsequently coated with gelatin, soaked again in 4% (v/v) formaldehyde-30% sucrose solution, and then sectioned at a thickness of 40 μm using a freezing microtome (HM 430; Thermo Fisher Scientific). The second and third sections were mounted for staining for every 3 consecutive sections. For the drug infusion study (*n* = 6), every second section was Nissl-stained with thionin solution, and every third section was stained with DAPI solution (Vectashield) for fluorescence microscopy. For the electrophysiological study (*n* = 8), every second section was stained with thionin solution, and every third section was stained with gold solution for myelin staining. Photomicrographs of each brain section were obtained using a microscope mounted with a digital camera (Eclipse 80i; Nikon). To accurately estimate the position of tetrodes, we reconstructed the configuration of tetrodes based on histology results, and then compared it with the actual configuration to match the numbering of the tetrodes (Voxwin, UK).

### Unit isolation

As previously described [32,70], all single units were manually isolated using a custom program (WinClust). Various waveform parameters (e.g., peak amplitude, energy, and peak-to-trough latencies) were used for isolating single units, but peak amplitude was the primary criterion. Units were excluded if more than 1% of spikes occurred within the refractory period (1 ms) and mean firing rates during the task epoch (from cue onset to response) were lower than 0.5 Hz.

### Single-unit analysis

#### Basic firing properties

Single units were grouped into bursting, regular-spiking, and unclassified neurons based on their autocorrelograms and interspike-interval histograms [71]. Specifically, cells were classified as bursting neurons if they met the following criterion:

max (autocorrelogram of 3–5 ms) > max (autocorrelogram of 0–50 ms)/2

Among the remaining neurons, those in which the mode of the interspike-interval histogram was less than 35 ms were classified as regular-spiking neurons. Neurons that did not belong to either group were categorized as unclassified neurons. Spike width was measured as the distance from peak to trough.

#### Trial filtering

All subsequent analyses described below were performed using correct trials only. An overview of the subsequent single-unit analysis process is presented in **S3 Fig**. To control for variability in choice latency (i.e., from cue offset to the end of choice response), we excluded trials where the latency exceeded 3 absolute median deviations of all correct trials. If a recording session had less than 5 correct trials in any of the 8 stimulus conditions, all units recorded in that session were excluded from further analysis.

#### Defining selective epoch

Firing rates were calculated within 50-ms time bins with increments of 10 ms. All subsequent analyses described below were performed on firing rates within the task epoch, defined as the 900-ms interval from the start of the sample phase to immediately preceding the end of the response phase. To identify a selective epoch in which firing rates were significantly different between *Boy* and *Egg* objects, we performed two-way repeated measures ANOVA (object identity and modality condition as 2 factors) in each time bin using trials from object conditions (2 objects with 3 modality conditions). The time bin with the largest effect size (η^2^) for the object identity factor was designated “peak selectivity time,” representing the moment when the firing rate difference between the 2 objects was maximal. The selective epoch was defined as having more than 5 consecutive time bins around the peak selectivity time, each with a *p*-value < 0.05 for the object identity factor.

#### Multiple linear regression

The following multiple linear regression models were used to describe firing patterns in relation to task-related conditions:

$$FR=\beta_{0}+\beta_{1}X_{1}+\beta_{2}X_{2}+\beta_{3}X_{3}+\beta_{4}X_{4}$$ (1)

$$FR=\beta_{0}+\beta_{1}X_{1}+\beta_{2}X_{2}+\beta_{3}X_{3}+\beta_{4}X_{4}+\beta_{5}X_{5},$$ (2)

where the dependent variable FR is the firing rate within the selective epoch described above. In the standard model (1), β_0_ is the constant term, β_1_ × *X*_1_ is the term for visual information of the preferred object, β_2_ × *X*_2_ is the term for auditory information of the preferred object, β_3_ × *X*_3_ is the term for visual information of the non-preferred object, and β_4_ × *X*_4_ is the term for auditory information of the non-preferred object. The independent variables (*X*) were binary-coded to reflect the existence of an image or sound for an object. For example, if a neuron was classified as a *Boy*-preferring object cell, *X*_1_ had a value of one in Boy-VA and Boy-V trials and zero in all other conditions. We found that the model with the 4 terms in the current study was optimal in quantifying responses to visual or auditory features of an object while minimizing multicollinearity problems between the terms. In the extended model (2), the term β_5_ × *X*_5_ was added to examine the influence of the response factor further. X_5_ had a value of one if a trial required a left-choice response and zero if it required a right-choice response. All trial conditions (VA, V, A, C) were used to estimate the regression model. β coefficients were standardized by z-scoring both dependent and independent variables prior to regression fitting. To dissociate neurons that were mainly modulated by choice responses (i.e., response cell) rather than object information, we quantified how much the model was improved by adding the response factor. Specifically, we subtracted the AIC (Akaike information criterion) for the extended model (2) from that for the standard model (1). If a neuron exhibited a significantly higher AIC difference, we concluded that most of its activity patterns were explained by the response factor and thus classified it as a response cell. The significance of the AIC difference was determined by comparison with the null distribution, obtained by shuffling trial conditions (shuffled 1,000 times; α = 0.01). Neurons with a selective epoch but not classified as response cells were categorized as object cells. To describe how object cells responded to different modality information, we examined regression coefficients in the standard model (1) using β_1_ and β_2_ to quantify how strongly an object cell responded to visual and auditory information, respectively, of a preferred object. We did not further examine regression coefficients for a non-preferred object (i.e., β_3_ and β_4_) (see **S6 Fig**). Neurons for which the difference between β_1_ and β_2_ was significantly higher or lower than the difference obtained after shuffling trial conditions were classified as visual or auditory cells, respectively (shuffled 1,000 times; α = 0.05, two-sided permutation test).

#### Rate modulation index

We calculated a “rate modulation index” (RMI) to quantify increases or decreases in a neuron’s firing rates in the multimodal condition relative to the unimodal condition. Firing rate differences between the multimodal and unimodal conditions were quantified using Cohen’s *d* as follows:

$$RMI=\frac{mean(VA)–mean(V\;or\;A)}{std(VA,V\;or\;A)}.$$

The index was calculated only in the modality conditions of the preferred object and was referred to as “VA–V” when it was calculated between multimodal and visual conditions and “VA–A” when it was calculated between multimodal and auditory conditions.

### Population decoding

A linear support vector machine (*sklearn*.*svm*.*SVC*, Python function), with a cost parameter set to 0.01, was used for population decoding. We confirmed that the support vector machine selected most of the trials (>95%) from various conditions as support vectors without bias in the given cost parameter. Population decoding was performed on rats in which at least 20 object cells were recorded across sessions (5 of 8 rats). Spikes were binned into 100-ms time bins within the task epoch (900-ms duration) and z-scored. Pseudo-populations of neurons were constructed in each rat as follows: Five trials for each object cell were subsampled for each of the 6 object conditions (2 objects × 3 modalities). Firing rates in the subsampled trials were horizontally concatenated to the pseudo-population. Thus, each pseudo-population had 30 rows (5 trials × 6 conditions) and *N* columns (or features), where *N* was the number of time bins (9) multiplied by the number of object cells. For modality-specific object decoding (**Fig 7A**), the entire subsampled dataset (30 samples) was used for both training and testing. One-versus-one classification was performed using stratified 5-fold cross-validation. For modality-invariant object decoding (**Fig 7D**), a binary classifier was trained using only unimodal trials and then tested with multimodal trials [72]. We did not perform cross-validation here since the training and test sets were completely separate. Subsampling, training, and testing were repeated 100 times in both decoding procedures, and the average of these repeated results was used as the representative value for each rat. A permutation test, performed by shuffling trial conditions, was used for significance testing (shuffled 1,000 times; α = 0.05). Confusion matrices (**Fig 7B** and **7E**) were constructed by averaging the results from all rats. Contributions to decoding performance (**Fig 7C** and **7F**) were measured using the permutation feature importance method. Specifically, after training the classifier, we selected all features from a given cell category (unimodal or crossmodal) and shuffled their rows (or trial labels) to break the relationship between the true label and selected features. The decrease in decoding accuracy after permutation was used as an indicator of how much the selected features contributed to decoding performance. Contribution to decoding was calculated as follows:

$$Contributions\;to\;decoding=\frac{Accuracy(baseline)–Accuracy(after\;permutation)}{Accuracy(baseline)+Accuracy(after\;permutation)}.$$

To measure the contribution of a single cell to decoding performance in a given category, we divided the value by the number of cells in that category within each rat.

### Quantification and statistical analysis

Data were statistically tested using custom-made codes written in MATLAB and Python. Student’s *t* test, analysis of variance (ANOVA), Wilcoxon sign-rank test, Chi-square test, and permutation test were used for statistical comparisons. A one-sample *t* test was used to verify that the behavioral performance was above the level of chance and that RMI values were significantly different from zero. One-way repeated measures ANOVA was implemented to compare behavioral results across modality conditions. Two-way repeated measures ANOVA was used to compare behavioral results (drug and modality condition as 2 factors), as well as to identify object-selective epoch (object and modality condition as 2 factors). Post hoc analyses were carried out using a *t* test with *p*-values corrected using the Holm–Bonferroni method. Wilcoxon signed-rank test was used to compare the regression coefficients, β_1_ and β_2_. An ordinary least squares method was used for both multiple and simple linear regression. The chi-square test was used to compare proportions. A permutation test was used for categorizing response-selective neurons and defining significance levels for population decoding accuracy. Unless otherwise indicated, the significance level was set at α = 0.05. Error bars indicate the standard error of the mean (SEM) unless stated otherwise.

## Supporting information

S1 Fig Nose-poking behaviors and their influence on performance; related to Fig 1. (**A**) Illustration of the task sequence for trials 20 through 35 in an example session. Here, trial start (0 on the y-axis) is the moment when the device becomes ready to accept the nose-poke behavior after the inter-trial interval has passed. The *Boy* (square) or *Egg* (triangle) object was pseudo-randomly presented regardless of whether the nose-poke was successful (>400 ms) on the previous attempt. Successful nose-poke attempts are marked with green lines. Prematurely withdrawn nose-pokes (<400 ms) are marked with red lines; the numbers above them indicate the duration of the rat’s nose-pokes in milliseconds. (**B**) Nose-poke failures did not have a significant influence on correctness. After rats failed to maintain nose-poke for 400 ms (red lines), they either experienced the same (blue) or a different (yellow) object on their next nose-poke attempt (top). There was no significant difference in correctness between the same and different stimulus situations (t_(5)_ = 0.27, *p* = 0.8, paired *t* test). Data are from sessions where rats (*n* = 6) performed above the learning criterion (correctness >75%). n.s., not significant. Source data are available in **S1 Data**.
(TIF)

S2 Fig Detailed behavioral data from electrophysiological experiments; related to Fig 3. (**A**) Choice latency (from cue offset to the end of choice response) data from 2 example sessions are shown, from rat #10 (left) and rat #13 (right). Similar latencies around 600 ms were marked if the rats had already completed their choice before the choice ports were fully opened and the sensors became available. Each dot indicates the choice latency of each trial. Although choice latency was more stable in rat #10 compared to rat #13, both rats completed most trials within a 600-ms choice latency. Trials were excluded for neural data analysis (red dots) if their choice latency was longer than the median + 3 × the median absolute deviation (dotted lines). (**B**) Average behavioral performance in all recording sessions. There were significant differences in correctness between conditions (F_(3,21)_ = 28.11, *p* < 0.0001; one-way repeated measures ANOVA). Performance in the multimodal condition (VA) was significantly higher than that in visual (V, t_(7)_ = 7.85, *p* = 0.0005) and auditory (A, t_(7)_ = 4.93, *p* = 0.0051; paired *t* test with Holm–Bonferroni correction) conditions. Performance in the control condition (C) was significantly higher than that in all the other conditions (control vs. multimodal, t_(7)_ = 4.22, *p* = 0.0078; control vs. visual, t_(7)_ = 12.51, *p* < 0.0001; control vs. auditory, t_(7)_ = 5.93, *p* = 0.0023; paired *t* test with Holm–Bonferroni correction). (**C**) Behavioral performance across recording sessions. Correctness was not significantly different across recording sessions (F_(4,28)_ = 1.58, *p* = 0.21; two-way repeated measures ANOVA), indicating that there was no additional learning after repeated sessions. There were significant differences between the conditions (F_(3,21)_ = 30.04, *p* < 0.0001), but the interaction effect between the recording session and condition factors was not significant (F_(12,84)_ = 1.56, *p* = 0.12; two-way repeated measures ANOVA). The dotted lines indicate chance level performance (50%). Data are presented as means ± SEM (*n* = 8; **p < 0.01, ***p < 0.001, ****p < 0.0001; n.s., not significant). Source data are available in **S1 Data**.
(TIF)

S3 Fig Analytic scheme for single-unit data. (TIF)

S4 Fig Response-selective firing patterns in the PER; related to Fig 4. (**A**) Example neuron showing selective firing patterns to a left choice response. Note that object *Boy* and C-L (control-left) conditions were associated with the same left choice response, whereas object *Egg* and C-R (control-right) conditions required the same right choice response. Spike density functions on the left showed increased firing rates when rats were producing a left-choice response. (**B**) Classification of the response cell in **A**. The AIC (Akaike information criterion) difference was calculated before and after adding the choice factor to the multiple linear regression model (see Methods). The histogram shows the AIC difference calculated from shuffled data (iterations = 1,000). The neuron was classified as a response cell because the cell’s actual AIC difference (red solid line) was significantly higher than the alpha level (red dotted line, *p* = 0.01). (**C**) Examples of object cells (left) and response cells (right) and their firing patterns to object and control conditions. Note that response cells showed overlapping firing patterns to both object and control conditions requiring the same choice response, but object cells did not. (**D**) Proportions of object and response cells within the PER. Numbers in parentheses denote the number of neurons. (**E**) Population selectivity plot for all response cells in the PER. Most of their selective epochs occurred during the response phase, in contrast to the sequential tiling of the entire task epoch by object cells shown in **Fig 4C**. The gray vertical line indicates the onset of the response phase. (**F**) Cumulative distributions of peak selectivity for the object (solid black line) and response (dotted gray line) cell categories. There were significant differences in peak selectivity time between categories (D = 0.46, *p* < 0.0001; Kolmogorov–Smirnov test). The peak selectivity time for most response cells occurred after 400 ms (i.e., the response phase). (**G**) Comparison of the duration of selectivity between object and response cells. The duration of selectivity for the response cell category was significantly longer compared with the object cell category (U = 832, ****p < 0.0001; Mann–Whitney U test); n.s., not significant. Source data are available in **S1 Data**.
(TIF)

S5 Fig Selective firing patterns of unimodal cells and their behavioral correlates; related to Fig 5. (**A**) Cumulative distributions of peak selectivity time for visual and auditory cells. Differences in peak selectivity time were marginally significant between the 2 categories of cells (D = 0.38, *p* = 0.053; Kolmogorov–Smirnov test). Peak selectivity for auditory cells was more likely to occur during the sample phase. The vertical gray line indicates the boundary between the sample and response phases. (**B**) Comparison of the duration of selective epochs between visual and auditory cells. The duration of selectivity for auditory cells was significantly longer than that for visual cells (U = 388.5, *p* = 0.03; Mann–Whitney U test). (**C**) Scatter plot of differences in coefficients (β_1_ –β_2_) showing relative performance in the visual condition, used to investigate the relationship between a neuron’s visual preference and the performance in the visual condition. Relative performance was obtained by dividing correctness in the visual (V) condition by correctness in the multimodal (VA) condition. Visual cells (cyan) were present regardless of the rat’s performance in the visual condition. There was no significant linear relationship between performance and the difference in coefficients (r = −0.071, *p* = 0.39). (**D**) Relationship between a neuron’s auditory preference and performance in the auditory condition. Relative performance was calculated by dividing correctness in the auditory (A) condition by correctness in the multimodal (VA) condition. Auditory cells (pink) were present irrespective of the rat’s performance in the auditory condition. No significant relationship was found between the difference in coefficients and relative performance in the auditory condition (r = 0.054, *p* = 0.51). Dotted black lines indicate the linear regression line, and the shaded areas represent the 95% confidence interval. #*p* = 0.053. *p < 0.05. n.s., not significant. Source data are available in **S1 Data**.
(TIF)

S6 Fig Regression coefficients for non-preferred objects; related to Fig 5. (**A**) Scatter plot showing the regression coefficients β_3_ and β_4_ for a non-preferred object (i.e., object conditions with lower firing rates). Cells were classified into visual (cyan) or auditory (pink) categories using the same procedure as in **Fig 5** but with β_3_ and β_4_ instead. β_3_ and β_4_ values for most neurons were around zero, indicating that they were not modulated by the modality information of non-preferred objects. (**B**) Proportions of visual and auditory cells classified using regression coefficients for the non-preferred object. Only a handful of neurons were classified as having a significant preference for the visual or auditory information of the non-preferred object. Numbers in parentheses indicate the number of cells. (**C**) Kernel density estimations of differences in coefficients for preferred (orange) and non-preferred (black) object conditions. For the preferred object, there were more neurons with extremely negative (auditory) or positive (visual) differences in coefficient values. However, the difference in coefficients for the non-preferred object was centered around zero, indicating no modulation by a specific sensory modality. The distributions of coefficient differences were significantly different between preferred and non-preferred object conditions (D = 0.19, *p* = 0.007; Kolmogorov–Smirnov test). **p < 0.01.
(TIF)

S7 Fig Anatomical locations of object cells along the anteroposterior axis of the PER and their peak selectivity time. Scatter plots of distance from bregma and the peak selectivity time of object cells were plotted separately for crossmodal (top), visual (middle), and auditory cells (bottom). There was no significant linear relationship between distance from bregma and peak selectivity time in all cell categories. The dotted black lines indicate the linear regression line, and the shaded areas represent the 95% confidence interval. n.s., not significant.
(TIF)

S8 Fig Firing rate modulations of crossmodal cells to different modality conditions; related to Fig 6. (**A**) Examples of crossmodal cells and their modulation patterns in different modality conditions. Spike density functions (i) and mean firing rates within the selective epoch (ii) demonstrate heterogeneous modulation patterns in crossmodal cells. In cell #17, mean firing rates were similar across all modality conditions of the preferred object (*Egg*), and RMI values were near zero (VA – V = −0.01, VA – A = 0.03). The firing rates of cell #18 were higher in the multimodal condition than in visual or auditory conditions, resulting in positive RMI values (VA – V = 0.39, VA – A = 0.37). On the other hand, firing rates for cell #19 were lower in the multimodal condition compared with both visual and auditory conditions, resulting in negative RMI values (VA – V = −0.6, VA – A = −0.67). (**B**) Scatter plot and histograms of VA – V and VA – A in crossmodal cells. Average VA – V (vertical gray line) and average VA – A (horizontal gray line) were not significantly different from zero (VA – V, t_(100)_ = 0.7, *p* = 0.49; VA – A, t_(100)_ = 0.49, *p* = 0.62; one-sample *t* test); n.s., not significant.
(TIF)

S9 Fig Relationship between multisensory suppression and behavior; related to Fig 6. (**A**) Scatter plots of RMI values (VA – V or VA – A) and relative performance, displayed separately for visual (left) and auditory (right) cells, used to determine whether multisensory suppression (i.e., negative VA – V or VA – A values) in visual or auditory cells is related to lower correctness in visual or auditory conditions. Relative performance was obtained by dividing correctness in the visual (V) or auditory (A) condition by correctness in the multimodal (VA) condition. Neither VA – V nor VA – A became more negative as the rat performance worsened in the visual or auditory condition. In both cell categories, no significant linear relationship was found between relative performance and RMI values (visual, r = 0.19, *p* = 0.42; auditory, r = 0.12, *p* = 0.57). Each dot indicates individual visual or auditory cells. (**B**) Scatter plots of RMI values (VA – V or VA – A) and neurons’ recorded sessions, displayed separately for visual (left) and auditory (right) cells, used to investigate whether multisensory suppression (i.e., negative VA – V or VA – A values) in visual or auditory cells is related to the novelty of the visual or auditory condition. Even on days 5 through 7, when rats were sufficiently acclimated to visual or auditory conditions, neurons exhibited negative VA – V or VA – A values. Therefore, it is unlikely that the suppression of activities in the multimodal condition is attributable to repetition suppression in the familiar multimodal condition. In both cell categories, no significant linear relationship was found between the recording session and RMI (visual, r = 0.03, *p* = 0.89; auditory, r = 0.16, *p* = 0.44). The dotted black lines indicate the linear regression line, and the shaded areas represent the 95% confidence interval. n.s., not significant.
(TIF)

S10 Fig Control analysis of modality-invariant object decoding; related to Fig 7. (**A**) If modality-invariant decoding were successful simply because an object always required the same choice response, we would expect to observe comparable decoding accuracy for control stimuli requiring the same choice response. We, therefore performed decoding of control stimuli based on visual and auditory object conditions using a linear support vector machine (SVM). The classifier was trained using the same dataset as used for modality-invariant decoding in **Fig 7D**. However, this time, we tested whether the same classifier could discriminate between 2 control stimuli that required the same choice response instead of the multimodal objects. (**B**) Comparison of decoding accuracies for multimodal objects (**Fig 7D**) and control stimuli. The decoding accuracy of the control stimuli was not comparable to that of the multimodal objects, suggesting that modality-invariant decoding was enabled by the object identity rather than the choice response. Decoding accuracy was significantly different between the 2 decoding methods (t_(4)_ = 3.61, *p* = 0.023, paired *t* test). The dotted black lines indicate the chance level of decoding accuracy obtained from surrogate data. Data are presented as means ± SEM (*p < 0.05). Source data are available in **S1 Data**.
(TIF)

S11 Fig Decoding object identities of unimodal trials based on multimodal trials; related to Fig 7. (**A**) Diagram summarizing multi-to-visual object decoding using a linear SVM. The SVM was trained with multimodal trials and then tested with visual trials. (**B**) Confusion matrix showing the average decoding accuracy of the classifier depicted in A (*n* = 5). The SVM trained with multimodal trials successfully decoded object identities from visual trials. (**C**) Diagram summarizing multi-to-auditory object decoding using a linear SVM. The SVM was trained with multimodal trials and then tested with auditory trials. (**D**) Confusion matrix showing the average decoding accuracy of the classifier depicted in C (*n* = 5). The SVM trained with multimodal trials successfully decoded object identities from auditory trials. ***p < 0.001, ****p < 0.0001. Source data are available in **S1 Data**.
(TIF)

S1 Table The number of PER neurons recorded in areas 35 and 36. (TIF)

S2 Table The number of visual, auditory, and crossmodal cells recorded in each rat. The numbers in the parenthesis indicate the percentage of the cell categories within each rat.
(TIF)

S1 Video The multimodal object-recognition task. (MP4)

S1 Data Source data. (XLSX)

## Abbreviations

AIC: Akaike information criterion
fMRI: functional magnetic resonance imaging
MUS: muscimol
PBS: phosphate-buffered saline
PER: perirhinal cortex
RMI: rate modulation index
SEM: standard error of the mean
SVM: support vector machine
